# Pbx loss in cranial neural crest, unlike in epithelium, results in cleft palate only and a broader midface

**DOI:** 10.1111/joa.12821

**Published:** 2018-05-23

**Authors:** Ian C. Welsh, James Hart, Joel M. Brown, Karissa Hansen, Marcelo Rocha Marques, Robert J. Aho, Irina Grishina, Romulo Hurtado, Doris Herzlinger, Elisabetta Ferretti, Maria J. Garcia‐Garcia, Licia Selleri

**Affiliations:** ^1^ Program in Craniofacial Biology Departments of Orofacial Sciences and Anatomy Institute of Human Genetics University of California San Francisco San Francisco CA USA; ^2^ Department of Cell and Developmental Biology Weill Cornell Medical College of Cornell University New York NY USA; ^3^ Department of Molecular Biology and Genetics Cornell University Ithaca NY USA; ^4^ Department of Physiology and Biophysics Weill Cornell Medical College of Cornell University New York NY USA; ^5^ Present address: Department of Morphology Piracicaba Dental School University of Campinas Sao Paulo Brazil; ^6^ Present address: Department of Urology New York University School of Medicine New York NY USA; ^7^ Present address: DanStem University of Copenhagen Bledamsvej 3B Copenhagen 2200 Denmark

**Keywords:** cleft lip/palate (CL/P), cleft palate only (CPO), Pbx, transcription factor, craniofacial, morphogenesis, skeleton, birth defect, tissue‐specific

## Abstract

Orofacial clefting represents the most common craniofacial birth defect. Cleft lip with or without cleft palate (CL/P) is genetically distinct from cleft palate only (CPO). Numerous transcription factors (TFs) regulate normal development of the midface, comprising the premaxilla, maxilla and palatine bones, through control of basic cellular behaviors. Within the *Pbx* family of genes encoding Three Amino‐acid Loop Extension (TALE) homeodomain‐containing TFs, we previously established that in the mouse, *Pbx1* plays a preeminent role in midfacial morphogenesis, and *Pbx2* and *Pbx3* execute collaborative functions in domains of coexpression. We also reported that *Pbx1* loss from cephalic epithelial domains, on a *Pbx2*‐ or *Pbx3*‐deficient background, results in CL/P via disruption of a regulatory network that controls apoptosis at the seam of frontonasal and maxillary process fusion. Conversely, *Pbx1* loss in cranial neural crest cell (CNCC)‐derived mesenchyme on a *Pbx2*‐deficient background results in CPO, a phenotype not yet characterized. In this study, we provide in‐depth analysis of PBX1 and PBX2 protein localization from early stages of midfacial morphogenesis throughout development of the secondary palate. We further establish CNCC‐specific roles of PBX TFs and describe the developmental abnormalities resulting from their loss in the murine embryonic secondary palate. Additionally, we compare and contrast the phenotypes arising from PBX1 loss in CNCC with those caused by its loss in the epithelium and show that CNCC‐specific *Pbx1* deletion affects only later secondary palate morphogenesis. Moreover*,* CNCC mutants exhibit perturbed rostro‐caudal organization and broadening of the midfacial complex. Proliferation defects are pronounced in CNCC mutants at gestational day (E)12.5, suggesting altered proliferation of mutant palatal progenitor cells, consistent with roles of PBX factors in maintaining progenitor cell state. Although the craniofacial skeletal abnormalities in CNCC mutants do not result from overt patterning defects, osteogenesis is delayed, underscoring a critical role of PBX factors in CNCC morphogenesis and differentiation. Overall, the characterization of tissue‐specific *Pbx* loss‐of‐function mouse models with orofacial clefting establishes these strains as unique tools to further dissect the complexities of this congenital craniofacial malformation. This study closely links PBX TALE homeodomain proteins to the variation in maxillary shape and size that occurs in pathological settings and during evolution of midfacial morphology.

## Introduction

Orofacial clefting represents the most common craniofacial birth defect (Dixon et al. 2011; Leslie & Marazita, 2013). Although not a major cause of mortality, this congenital malformation imposes a psychological and financial burden on affected individuals, their families and society (Zeytinoglu & Davey, 2012). The phenotypic spectrum of this disorder ranges from microform lip defects to overt clefting that can involve the primary and/or secondary palate (Kim et al. 2010; Howe et al. 2015). It is also believed that cleft lip with or without cleft palate (CL/P) is genetically distinct from cleft palate only (CPO; Lidral & Moreno, 2005). Whereas facial clefting can be associated with syndromic conditions with well characterized genetic mutations, non‐syndromic or isolated clefting involves complex interactions between genetic and environmental risk factors (Marazita, 2012; Leslie & Marazita, 2013). Epidemiologic and genome‐wide studies of affected groups have led to the identification of putative genetic determinants of facial clefting, some of which have been evaluated in mouse models (Gritli‐Linde, 2012). Conversely, molecular analyses of mouse‐mutants generated by gene targeting have helped the discovery of new regulatory networks and candidate genes for human clefting. As a result, numerous factors have been found to contribute to development of the lip, primary and secondary palates (Kousa & Schutte, 2016; Tam et al. 2016).

Development of both primary and secondary palate requires cranial neural crest cell (CNCC) specification, migration, proliferation and differentiation, a process that is instructed by genetic networks whose regulatory topology is becoming increasingly well defined (Martik & Bronner, 2017). In mammals, growth of the secondary palate follows a stereotypical pattern (Ferguson, 1988; Lan et al. 2015). The murine secondary palate becomes visible at E11.5 as two parallel shelves that are rostro‐caudally oriented and grow vertically downwards from the maxilla on either side of the tongue. Concomitantly, vertical outgrowth of the shelves is also accompanied by growth along the anterior‐posterior (A–P) axis of the midface (comprising premaxilla, maxilla and palatine bones). By E14.5, the shelves further grow towards the midline and then adopt a horizontal disposition dorsal to the tongue. Subsequently, they fuse in the sagittal plane as well as with the nasal septum dorsally and the caudal border of the primary palate rostrally. Development of the secondary palate involves various basic cellular behaviors including proliferation, migration, apoptosis and differentiation (Cox, 2004; Bush & Jiang, 2012). During morphogenesis of the upper lip, primary palate and other organ systems, these behaviors have been shown to operate, at least in part, under mechanisms that are controlled by Pbx homeodomain transcription factors (TFs; Capellini et al. 2011; Ferretti et al. 2011).

Mammalian *Pbx* genes (*Pbx 1,2,3,4*) encode Three Amino‐acid Loop Extension (TALE) homeodomain‐containing TFs (Moens & Selleri, 2006; Longobardi et al. 2014), which play integral roles in the development of many organs in the mouse, including axial and appendicular skeleton, lung, heart, pancreas, spleen, kidney (Selleri et al. 2001; Kim et al. 2002; Capellini et al. 2006, 2008, 2010; Stankunas et al. 2008; Koss et al. 2012; Li et al. 2014; Hurtado et al. 2015; McCulley et al. 2018) and craniofacial complex (Ferretti et al. 2011). We reported that, among all the *Pbx* constitutive compound mutants generated, only *Pbx1* ^*−/−*^ *;Pbx2* ^*+/−*^ (*Pbx1/2*) and *Pbx1* ^*−/−*^ *;Pbx3* ^*+/−*^ (*Pbx1/3*) mutant embryos show fully penetrant CL/P and *Pbx1* ^*+/−*^ *;Pbx2* ^*+/−*^ *;Pbx3* ^*+/−*^ mutants die at birth with CPO. In contrast, single constitutive mutants for *Pbx1* die at midgestation but do not exhibit orofacial clefting on a mixed genetic background (Selleri et al. 2001). Notably, constitutive loss of *Pbx2* alone does not yield detectable phenotypes in the mouse (Selleri et al. 2004) and single constitutive loss of *Pbx3* results in postnatal lethality due to respiratory failure without craniofacial defects (Rhee et al. 2004). Thus, we established that Pbx1 plays a preeminent role in the development of the midface and other organ systems, and *Pbx2* and *Pbx3* execute collaborative functions in domains of coexpression (Capellini et al. 2006, 2008, 2010; Ferretti et al. 2011; Koss et al. 2012; Golonzhka et al. 2015). Furthermore, we described that *Pbx1* conditional loss from *Foxg1*‐positive (and *Crect*‐positive) cephalic epithelial domains, on a *Pbx2*‐ or *Pbx3*‐deficient background, results in CL/P via perturbation of regulatory networks that control apoptosis and epithelial‐to‐mesenchymal transition (EMT) at the seam wherein the frontonasal processes fuse with the maxillary process (Ferretti et al. 2011; Losa et al. 2018). In contrast, we reported that on a mixed genetic background, *Pbx1* inactivation in *Wnt1*‐positive CNCC‐derived mesenchyme on a *Pbx2*‐deficient background does not yield CL but CPO (Ferretti et al. 2011). However, the anatomical, cellular and molecular basis of the latter craniofacial defects has not yet been characterized.

Here, we provide detailed analyses of the tissue‐specific roles of PBX TFs in the murine CNCC and of the abnormalities resulting from PBX loss in the embryonic secondary palate. As opposed to the lip and primary palate defects associated with *Pbx1* epithelial loss, CNCC‐specific loss disrupts secondary palate morphogenesis and organization of the craniofacial skeletal complex. Together, our findings highlight distinct tissue‐specific roles and iterative functions of PBX TFs in the coordinated development of the midfacial complex.

## Materials and methods

### Mice

The mutant alleles used in this study have been published previously and include: the *Pbx1* constitutive and conditional mutant alleles (Selleri et al. 2001; Koss et al. 2012), the *Pbx2* constitutive knock‐out allele (Selleri et al. 2004), the *Wnt1‐Cre* transgene (Lewis et al. 2013) and the *Foxg1‐Cre* knock‐in allele (Hebert & McConnell, 2000). The *Foxg1‐Cre* deleter line was maintained on a pure Swiss Webster genetic background and the *Wnt1‐Cre* transgenic line was kept on a mixed Swiss Webster/C57Bl6 genetic background.

Mouse embryos were harvested at the appropriate developmental stage from pregnant dams. The presence of a vaginal plug was assessed as embryonic day 0.5 (E0.5). Mutant and control embryos were either somite‐matched (E10.0, E11.5) or matched by crown‐rump length (E12.5 onwards). Dams were euthanized by CO_2_ administration followed by cervical dislocation as approved by Weill Cornell institutional and UCSF IACUC protocols.

### Histological analysis

Embryos were harvested and fixed O/N at 4 °C in 0.1 m phosphate buffer (PB) containing 4% (w/v) paraformaldehyde (PFA). Fixation time varied from O/N (minimum) for E10.5–E12.5 embryos to longer times as appropriate for larger embryos. Embryonic tissues were dehydrated in serial alcohols before clearing in a proprietary clearing agent (Histoclear‐National Diagnostics) followed by paraffin wax embedding. After sectioning, paraffin‐embedded sections were dewaxed, re‐hydrated, stained with hematoxylin before being counterstained with eosin. Images were obtained using a Zeiss AxioPlan upright microscope.

### Scanning electron microscopy

Embryos were harvested and fixed at 4 °C in 0.1 M phosphate buffer containing 4% (w/v) paraformaldehyde (PFA) for several days. Paired samples were then immersed in 2.5% glutaraldehyde, 4% paraformaldehyde, 0.02% picric acid in 0.1 M phosphate buffer O/N. Post fixation samples were treated with aqueous osmium‐tetroxide ferricyanide solution (1% OsO_4_‐1.5%K‐ferricyanide) O/N. After dehydration through a graded ethanol series and critical point drying through liquid CO2, samples were mounted on aluminum stubs and sputter coated with gold‐palladium. Samples were imaged using a ZEISS LEO 1550 Scanning Electron Microscope.

### Immunofluorescence antibody staining

Embryos were harvested in cold phosphate‐buffered saline (PBS) and briefly fixed in 0.1 m phosphate buffer containing 4% PFA at 4 °C according to gestational age: (E10.5: 45 min; E11.5: 1 h; E12.5: 1.5 h; E13.5: 2 h). After washing in cold PBS, embryos were incubated O/N at 4 °C in 30% Sucrose/PBS. Embryos were then embedded in a 1 : 1 mixture of OCT and 30% Sucrose.

After cryosectioning at a standard thickness of 10 μm, slides were washed with PBS and treated with a solution of sodium borohydride (NaBH_4_) in PBS depending upon gestational stage: (E10.5/E11.5: 0.01% solution for 10 min; E12.5: 0.1% solution for 5 min; E13.5: 0.1% solution for 5 min). After three washes in PBS, slides were immersed in TSP Buffer (0.5% Triton, 0.1% Saponin, 1× PBS) for 10 min before blocking in 1% Normal Donkey Serum (NDS) in TSP for 1 h at 37 °C with gentle rocking. Depending upon gestational age, primary antibodies were applied at the following concentrations and incubation times at 37 °C in 1% NDS/TSP: anti‐active Caspase‐3 pAb (Promega), 1 : 200 for 1 h; anti‐Pbx1 (Cell Signaling) E10.5/E11.5, 1 : 100 for 1 h; E12.5, 1 : 75 for 2 h; E13.5, 1 : 50 for 2 h); anti‐Pbx2 (Santa‐Cruz) E10.5, 1 : 100 for 1 h; E11.5/E12.5, 1 : 50 for 2 h. After washing, fluorescent secondary antibody AlexaFluor™647 (Invitrogen‐ThermoFisher Scientific) was applied, for 1 h at 37 °C in 1% NDS/TSP, at a dilution of 1 : 200. Slides were then washed and counterstained with DAPI at 1 : 5000 for 10 min prior to coverslipping.

### MicroCT imaging and cephalometric analysis

Fixed, unstained E18.5 mouse embryos were scanned at 18 μm resolution using a Zeiss Xradia Versa 520 XRM (Cornell University BRC Imaging Facility; X‐ray source 80 kV/7W, LE1 filter). 3D reconstructions of the data and rotational movies (200 frames/20 fps) were rendered using osirix software (Pixmeo SARL). The palatal region and individual bones were isolated with the automated osirix 3D Segmentation tool followed by manual refinement (Rosset et al. 2004). The osirix Crop tool was used to remove palatal structures on the left side in ‘cropped’ views. Manual 2D bone measurements were obtained using the osirix Length tool. For 3D measurements, landmarks were manually placed in osirix, and excel was used to compute distances between the XYZ coordinates of each landmark. All measurements were normalized to the rostral‐caudal length of the skull and made relative to the wild type average. *P*‐values were calculated using two‐tailed Student's *t*‐tests with unequal variance.

### Cell proliferation assays

The Click‐iT EDU reaction assay was carried out according the manufacturer's instructions (ThermoFisher Scientific). Pregnant dams were administered EdU at 50 μg kg^−1^ by intraperitoneal injection before sacrifice. Sacrifice was carried out after 30 min, 45 min or 1 h for E11.5, E12.5 and E13.5 stages, respectively. Embryos were processed for cryosectioning as for immunofluorescence antibody staining. After sectioning, slides were washed and permeabilized with 0.5% Triton X‐100 in PBS. The Click‐iT™ reaction was prepared and applied to slides as per the manufacturer's instructions. After development, slides were washed and counterstained with DAPI at 1 : 5000 for 10 min, followed by coverslipping. EdU‐positive cells were counted from three embryos per each time‐point examined. Three sections, corresponding to anterior, middle and posterior secondary palate, were examined for each embryo. Quantification of the percentage of EdU‐positive cells vs. total number of cells present in each palatal domain was performed using algorithms generated with matlab software (MathWorks). All cells within enclosed areas were counted for all slides analyzed corresponding to anterior, middle and posterior domains. The percentage of proliferating cells at each developmental stage examined was calculated by dividing the number of EdU‐positive cells by the total number of counted cells (400–700) on each section.

### Apoptosis assays

Embryos were harvested in cold PBS and cryopreserved as described previously (see Immunofluorescence Antibody Staining). Anti‐active Caspase‐3 primary antibody (Promega Corp.) was applied at 1 : 200 in 1% NDS/TSP for 1 h 37 °C with rocking. After washing, fluorescent secondary antibody, AlexaFluor™647 (Invitrogen‐ThermoFisher Scientific) was applied, also at a dilution of 1 : 200. Slides were then washed and counterstained with DAPI at (1 : 5000) for 10 min, followed by coverslipping. Three pairs of embryos (control and mutant) were analyzed for each time point (E11.5–E13.5). Palatal sections were categorized as anterior, middle or posterior depending on their location and stereotypical morphology. Fluorescent images of palatal sections were obtained using a Hamamatsu C4742‐95 camera (Hamamatsu Corp.), mounted on a Nikon Eclipse TE200 microscope (Nikon Corp.). Quantification of the percentage of Caspase‐three positive cells vs. total number of cells present in each palatal domain was performed using algorithms generated with matlab software, as described above for the Cell Proliferation Assay. The total number of cells counted on each section was 400–700.

### Preparation of *in situ* hybridization probes

Murine cDNA probes for *Msx1* (Hill et al. 1989) and *Barx1* (Miletich et al. 2005) were obtained from Drs Richard Maas and Paul Sharpe, respectively. For murine *Tbx22* and *Alpl*, a 1250‐bp cDNA clone corresponding to nucleotides 307–1547 of RefSeq NM_145224 and a 1043 bp cDNA clone corresponding to nucleotides 1380–2422 of RefSeq NM_001287172, respectively, were TA‐cloned from E14.5 C57Bl6 cDNA and used to transcribe DIG‐labeled antisense probes. For murine *Shox2*, a 660‐bp cDNA sequence within the *Shox2* RefSeq NM_013665.1 was PCR‐amplified with primers appended with the recognition sequence for T7 RNA polymerase. The purified PCR product was subsequently used as a template for *in vitro* transcription and DIG labeling. All probes were labeled using a commercially available *in vitro* transcription kit (Roche), following the manufacturer's instructions.

### *In situ* hybridization

All embryos were dissected into cold PBS and fixed O/N in 4% PFA/PBS. Embryonic heads were then dehydrated through a methanol‐PBT series, with gentle rocking at room temperature. Embryos were stored at −20 °C in 100% methanol until processing.

After bleaching in 6% hydrogen peroxide for 20 min, embryos were treated with Proteinase K (20 μg mL^−1^) for 10 min and then quenched with glycine solution (2 mg mL^−1^) before being washed twice in PBT. Embryos were then re‐fixed in 4% PFA/0.2% glutaraldehyde in PBT for 20 min before being washed twice in PBT. Embryos were then transferred to a prehybridization solution for 1 h at 70 °C. Riboprobes were added (1 μg mL^−1^) for incubation overnight at 70 °C. Embryos were subsequently washed and treated with anti‐digoxigenin‐AP antibody (Roche) at a concentration of 1 : 2000 to 1 : 4000 (dependent on probe) and incubated overnight at 4 °C with rocking. Embryos were then washed extensively in TBST buffer at room temperature, prior to colorimetric detection with BM‐Purple Chromogenic Reagent (Roche). Lastly, embryos were washed and post‐fixed with 4% PFA/0.2% glutaraldehyde. Embryos were imaged using a Leica MZ75 Stereo Zoom Microscope and an Omax A3590U 9MP digital camera. A minimum of three pairs (WT‐mutant) were used to assess the palatal expression pattern of each reported probe.

## Results

### **Dynamic spatiotemporal localization** of PBX1 and PBX2 in the developing primary and secondary palate

Previous studies have reported widespread expression of genes encoding TFs of the PBX family in the developing rodent embryo (Roberts et al. 1995), including the nascent secondary palate (Schnabel et al. 2001). Because mouse mutants with compound constitutive loss of *Pbx1* and *Pbx2* (*Pbx1* ^−/−^;*Pbx2* ^+/−^) display craniofacial phenotypes including CL/P (Ferretti et al. 2011), we analyzed in detail the patterns of PBX protein localization within the facial prominences, as well as primary and secondary palate. By immunofluorescent antibody (Ab) staining, we detected PBX1 and PBX2 protein products in the cephalic epithelium and mesenchyme from early stages of facial development (E10.5) (Supporting Information Figs S1 and S2). At the level of the dorsal maxillary process (MxP), both PBX1 and PBX2 are present at high levels in the epithelium and throughout the underlying mesenchyme (Figs S1E,F and S2E,F), whereas in the ventral MxP they are localized to the more rostral epithelium and in a medially restricted band in the underlying mesenchyme (Figs S1H,I and S2H,I).

**Figure 1:**
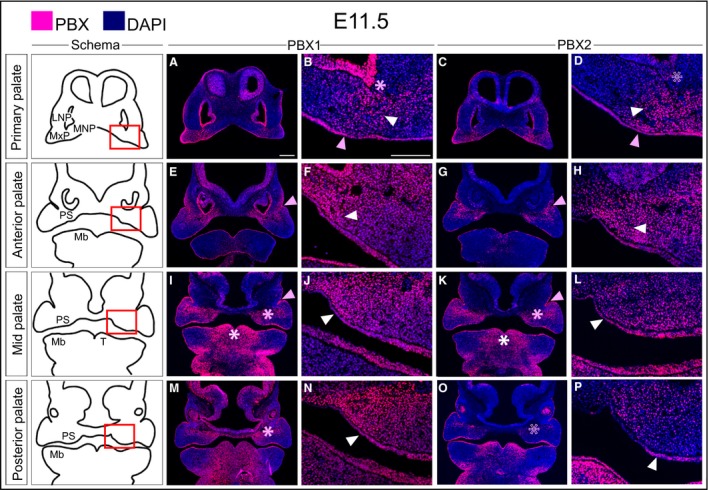
Localization of PBX1 and PBX2 in the developing midface at E11.5. Representative micrographs of immunofluorescence with PBX1‐ and PBX2‐specific antibodies on coronal sections through the primary and secondary palate. Cartoons of primary palate and secondary palate (anterior, mid, and posterior) are shown in left‐most column. PBX1 and PBX2 proteins, fuchsia signal; DAPI highlighting the nuclei, blue. Primary palate: (A–D) PBX1 and PBX2 are localized to the midfacial mesenchymal core where MNP, LNP and MxP converge (white arrowhead), as well as in the overlying epithelium (pink arrowhead). High levels of PBX1 in epithelium of the olfactory pit (pink asterisk in B) with low levels of PBX2 (empty pink asterisk in D). Anterior secondary palate: (E–H) Mesenchymal localization of PBX1 extends as a band from the naso‐lacrimal groove (NLG; pink arrowhead in E) into the palatal shelf primordium (white arrowhead in F) with PBX2 showing similar, albeit restricted, pattern (G,H). Both PBX1 and PBX2 also localize to the epithelium of the developing palatal shelf (F,H). Middle secondary palate: (I–L) Mesenchymal localization of both PBX1 and PBX2 extends medially from the NLG (pink arrowhead in I,K) into the palatal primordia in compressed band (pink asterisk in I,K) and is maintained in the mesenchyme and epithelium of the palatal shelf primordia (white arrowhead in J,L). PBX1 and PBX2 are localized to the tongue mesenchyme and epithelium (white asterisk in I,K). Posterior secondary palate: (M–P) PBX1 extends from the maxillary primordia into the palatal shelf primordia (pink asterisk in M), with sparser PBX2 mesenchymal expression (empty pink asterisk in O). In the overlying epithelium PBX1 is uniform (white arrowhead in N), with PBX2 restricted to the ventral‐lateral epithelium of the shelf (white arrowhead in P). LNP, lateral nasal process; Mb, mandible; MNP, medial nasal process; MxP, maxillary process; PS, palatal shelf; T, tongue. Magnification: columns 3 and 5, higher magnifications of the fields within red box in schemata of column 1. Scale bar: 200 μm.

By E11.5, during primary palate morphogenesis and fusion, PBX1 is evident in the cephalic epithelium and exhibits the highest levels at the lambdoidal junction where the medial nasal process (MNP), lateral nasal process (LNP) and MxP converge (Fig. 1A–D). These findings corroborate earlier reports on the requirement for *Pbx1* and *Pbx2* cephalic epithelial expression during lip and primary palate formation (Ferretti et al. 2011). At the site of primary palate fusion, PBX1 also shows a mesenchymal enrichment (Fig. 1A,B). Along the A–P axis of the developing secondary palate, PBX1 is present in a restricted band that extends medio‐laterally from the forming palatal shelf (Fig. 1E,F,I,J) to the epithelial invagination that will give rise to the naso‐lacrimal groove (de la Cuadra‐Blanco et al. 2003). More posteriorly, this band of mesenchymal PBX1 localization appears to become dorsally restricted and to divide the palatal field into dorsal and ventral (presumptive nasal and oral) domains (Fig. 1M,N). At this stage, PBX2 largely overlaps, albeit not as widely (Fig. 1C,D,G,H,K,L,O,P), the PBX1 spatial pattern.

**Figure 2:**
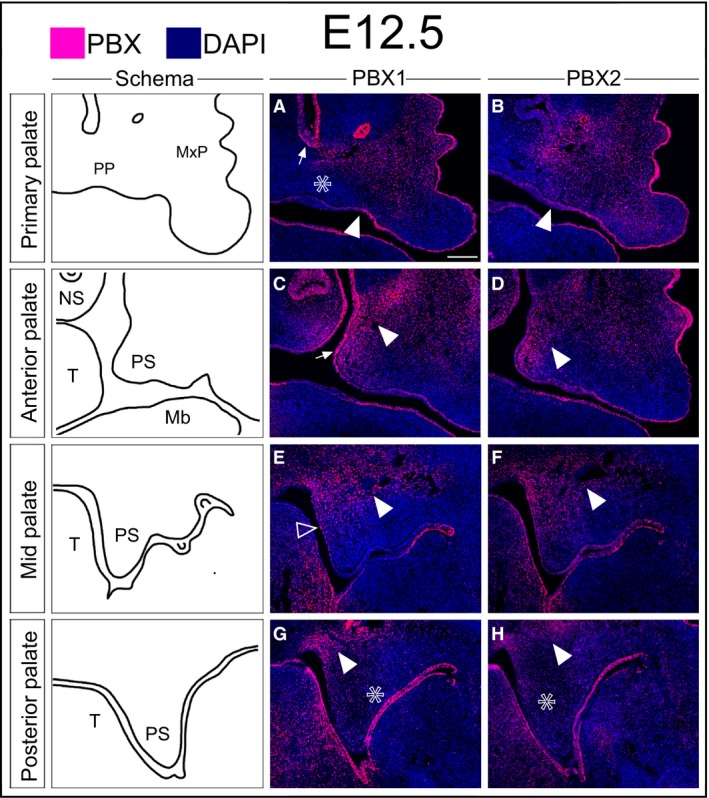
Localization of PBX1 and PBX2 in the developing midface at E12.5. Immunofluorescent detection of PBX1 and PBX2 (fuchsia signal) on coronal sections through the primary and secondary palate (DAPI, highlighting nuclei in blue). Cartoons of primary palate and secondary palate (anterior, mid, posterior) in left‐most column. Primary palate: (A,B) PBX1 and PBX2 in the oral epithelium, with higher levels lateral to the incisor bud (white arrowhead in A,B) throughout the surface cephalic ectoderm. PBX1 present in the mesenchyme of the primary palate but excluded from the condensation adjacent to the incisor bud (white empty asterisk in A). PBX1 also localized to the nasal epithelium (white arrow in A). PBX2 detectable throughout the anterior midfacial mesenchyme (B). Anterior secondary palate: (C,D) PBX1 levels higher in dorsal aspect of the MxP, palatal shelf proper (white arrowhead in C), and overlying epithelium (white arrow in C). PBX2 present at comparable levels in the palatal shelf and at lower levels in MxP (D). Middle secondary palate: (E,F) Mesenchymal localization of PBX1 and PBX2 confined to the dorsal‐most aspect of the palatal shelf and MxP (white arrowhead in E,F). In the epithelium, low levels of PBX1 restricted to the medial domain of the palatal shelf (white empty arrowhead in E) with broader distribution of PBX2 (F). Posterior secondary palate: (G,H) Weak mesenchymal levels of PBX1 and PBX2 in ventral palatal shelf mesenchyme (white empty asterisk in G,H), with band of higher PBX1 signal in MxP dorsal to shelf proper (white arrowhead in G, H). Epithelial localization of PBX1 and PBX2 confined to the oral side (G,H). Mb, mandible; MxP, maxillary process; NS, nasal septum; PP, primary palate; PS, palatal shelf; T, tongue. Scale bar: 200 μm.

At E12.5, PBX1 is excluded from the medial aspect of the primary palate epithelium and is largely confined to the region lateral to the developing incisor bud, where mesenchymal PBX1 is localized to the maxilla and extends medially into the dorsal aspect of the primary palate (Fig. 2A). At the level of the anterior secondary palate, PBX1 is present at high levels along the medial aspect of the palatal shelf (both epithelium and mesenchyme) and extends dorso‐laterally into the maxillary primordium (Fig. 2C). In the mid‐secondary palate, PBX1 is confined to the mesenchyme of the dorsal aspect of the maxilla and palatal shelf (Fig. 2E). The mesenchyme of the posterior secondary palate exhibits a similar pattern of PBX1 mesenchymal localization (Fig. 2G). As with earlier stages, in these domains PBX2 shows a similar, although weaker and more diffuse, pattern as PBX1 (Fig. 2B,D,F,H). This finding is consistent with our previous genetic studies reporting that PBX1 plays prominent roles in directing development of multiple organ systems including limb, axial skeleton, visceral organs, brain and midface, whereas PBX2 functions collaboratively with PBX1 in domains of coexpression (Capellini et al. 2006, 2008, 2010; Ferretti et al. 2011; Koss et al. 2012; Golonzhka et al. 2015; reviewed in Capellini et al. 2011). Accordingly, we focused our subsequent studies on PBX1 localization at later gestational stages.

In particular, IF analysis at E13.5 demonstrates that although PBX1 is absent from the premaxillary condensation of the primary palate, it continues to be localized to spatial domains critically involved in secondary palate morphogenesis (Supporting Information Fig. S3A–D). For example, PBX1 shows marked enrichment within the medial aspect of the palatal shelf along its entire A–P axis (Fig. S3B–D). Conversely, PBX1 appears to be down‐regulated in lateral aspects of the more posterior maxilla. Similarly, whereas epithelial PBX1 levels are high in the anterior domain of the primary and secondary palate, they are reduced in the epithelium of the more posterior secondary palate (Fig. S3A–D). In summary, our findings establish that PBX1 and PBX2 proteins are localized across the primary palate and the entire A–P axis of the secondary palate, in epithelial and CNCC‐derived mesenchymal domains that are critically involved in primary and secondary palatal morphogenesis. The dynamic spatiotemporal pattern of *Pbx1* and *Pbx2* expression suggests that these genes play tissue‐specific and iterative roles during critical phases of midfacial morphogenesis.

### Strikingly different phenotypes result from loss of *Pbx* genes in cephalic epithelium or cranial neural crest‐derived mesenchyme

Development of both primary and secondary palate requires CNCC specification, migration, proliferation, patterning and differentiation through reciprocal interactions between mesenchyme and overlying pharyngeal ectoderm (Ferguson, 1984; Minoux & Rijli, 2010). Using compound *Pbx1/Pbx2* constitutively mutant alleles (*Pbx1* ^*−/−*^ *;Pbx2* ^*+/−*^) that develop fully penetrant CL/P, we previously reported the absence of detectable differences in the expression domains of *Tfap2α* (Ferretti et al. 2011) in the nascent midfacial prominences of E10.0 *Pbx1* ^*−/−*^ *;Pbx2* ^*+/−*^ mutants compared with *Pbx1* ^*+/−*^ *;Pbx2* ^*+/−*^ control embryos, suggesting that loss of PBX factors does not affect early craniofacial development. In addition, we show here that *Msx1* CNCC‐specific expression is also grossly unchanged in E10.0 *Pbx1* ^*−/−*^ *;Pbx2* ^*+/−*^ constitutive mutant embryos vs. controls (Supporting Information Fig. S4). Collectively, these results indicate that equivalent populations of CNCC are present at the onset of midfacial morphogenesis in *Pbx* compound mutants and controls and that craniofacial defects associated with PBX loss are not a consequence of perturbed CNCC allocation or migration.

To inactivate *Pbx1* conditionally in the surface cephalic epithelium (SCE), we utilized the *Foxg1‐Cre* knock‐in allele (Hebert & McConnell, 2000), which directs Cre‐mediated recombination in the cranial epithelium and telencephalon. In contrast, to excise *Pbx1* from the CNCC‐derived mesenchymal population of the developing midface, we employed a *Wnt1‐Cre* transgene (Lewis et al. 2013), which exhibits activity in the premigratory CNCC population. Although CL/P was fully penetrant in *Pbx1* ^*fl/fl*^ *;Foxg1* ^*Cre/+*^ epithelial mutants with a single exception (hereafter referred to as epithelial mutants), we used a *Pbx2* sensitized genetic background, as reported (Ferretti et al. 2011), to generate a robust and fully penetrant midfacial phenotype in *Pbx* CNCC mutants. Thus, all CNCC‐specific *Pbx1* mutant embryos analyzed in this study were heterozygous for a *Pbx2* constitutive null allele on a mixed genetic background (*Pbx1* ^*fl/fl*^ *;Pbx2* ^*+/−*^ *;Wnt1‐Cre* ^*Tg/+*^ embryos; hereafter referred to as CNCC mutants). We validated the activity of both *Cre* mouse strains via IF detection of PBX1 protein in either epithelial or CNCC mutant embryos (Supporting Information Figs S5A′–D′ and S6A′–D′). We observed complete epithelial or CNCC mesenchymal loss of PBX1 in both mutant genotypes.

**Figure 3:**
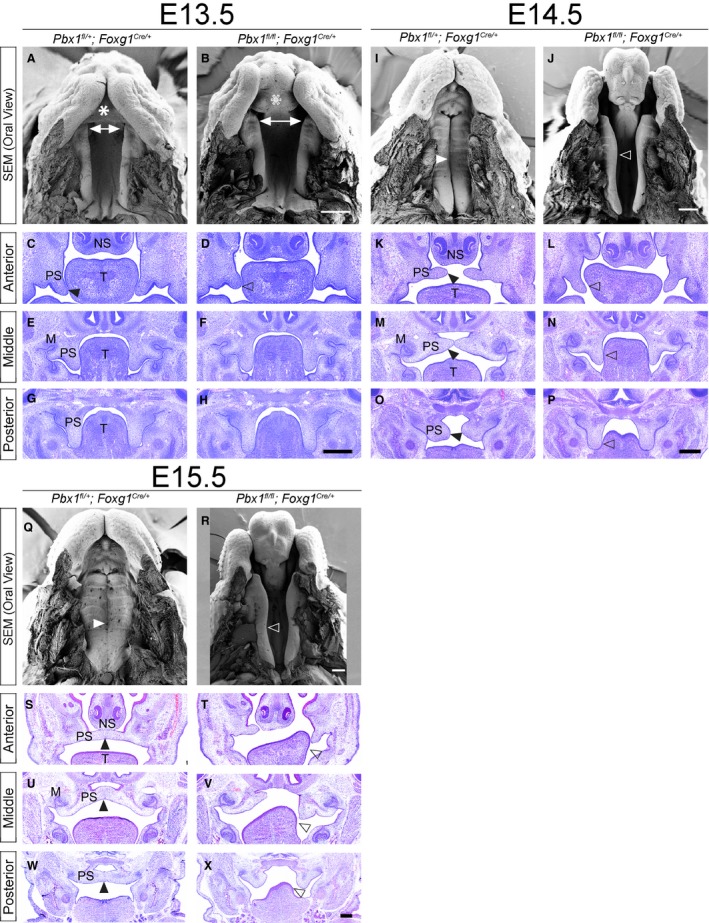
Epithelial loss of PBX1 results in clefting of the primary and secondary palate (CL/P). Scanning electron micrographs (SEM; top) and hematoxylin & eosin (H&E) stained coronal sections (bottom) of embryonic palate and oral cavity from E13.5–E15.5. Representative control: *Pbx* ^*fl/+*^ *;Foxg1* ^*Cre/+*^ (left) and mutant: *Pbx1* ^*fl/fl*^ *;Foxg1* ^*Cre/+*^ (right) embryos for each time point. E13.5: SEM shows dysmorphic primary palate in mutant (empty white asterisk in B) as compared with control (white asterisk in A). Anterior palatal shelves appear to be more widely spaced in mutant (white double‐headed arrows in A,B). H&E illustrates mild outgrowth defects of anterior secondary palatal shelves in mutant (empty black arrowhead in D) than in control (black arrowhead in C). Morphology of mutant middle and posterior secondary palatal shelves is otherwise comparable to that of control. E14.5: SEM highlights bilateral clefting of lip and primary palate in mutant (J) vs. control (I). Secondary palatal shelves remain unelevated and have not made contact medially (open white arrowhead in J), whereas in control they have elevated and made contact (white arrowhead in I). H&E sections show failure to elevate secondary palatal shelves in mutant as a potential consequence of aberrant trapping of shelves by tongue (empty black arrowhead in L,N,P), as compared with control (black arrowhead in K,M,O). E15.5: SEM shows complete clefting of secondary palate in mutant (open white arrowhead in R) vs. control (white arrowhead in Q). H&E illustrates that mutant secondary palatal shelves are cleft along entire A–P axis with aberrant contact with tongue (open black arrowhead in T,V,X), as compared with control (black arrowhead in S,U,W). M, molar tooth bud; NS, nasal septum; PS, palatal shelf; T, tongue. Scale bar: 400 μm.

To characterize the tissue‐specific functions of PBX1 during morphogenesis of the primary and secondary palate, we employed scanning electron microscopy (SEM) and histological analyses to compare and contrast the phenotypes resulting from epithelial (*Pbx1* ^*fl/fl*^ *;Foxg1* ^*Cre/+*^) and CNCC‐derived mesenchymal (*Pbx1* ^*fl/fl*^ *;Pbx2* ^*+/−*^ *;Wnt1‐Cre* ^tg/+^) *Pbx1* loss. Our gross morphological analysis demonstrated that CNCC mutants display CPO with full penetrance and comparable expressivity. In contrast, the epithelial mutants exhibit clefting phenotypes of the lip and primary palate with variable expressivity, confirming and expanding previously published results on a *Pbx2*‐sensitized background (Ferretti et al. 2011; summarized in Supporting Information Fig. S7A–E and Table S1). Briefly, the 21 epithelial mutants examined exhibited either normal lip and primary palate, with clefting of the secondary palate (33%) or unilateral or bilateral clefting of the lip and primary palate accompanied by clefting of the secondary palate (62%). A single epithelial mutant displayed normal primary and secondary palate with unilateral cleft lip (5%). In all cases except for one the phenotype of the lip and primary palate were correlated. Scanning electron microscopy (SEM) of the developing oral cavity in epithelial mutants from E13.5 to E15.5 revealed dysmorphic primary palate in conjunction with cleft lip (Fig. 3, top rows for all gestational days). Specifically, at E13.5, the primary palate appears broader, consistent with lack of fusion of the facial prominences. For the secondary palate, SEM also revealed a widening of the gap intervening between the anterior‐most aspects of the palatal shelves, indicative of an overall broadening of the midface (Fig. 3A–H). Both SEM and histology on serial coronal sections demonstrated relatively comparable morphologies of the secondary palate in controls and epithelial mutants, even though E14.5 mutants failed to elevate the palatal shelves (Fig. 3I–P). Histology at E14.5 and E15.5 suggests that failure to elevate can be unilateral or bilateral and can be at least in part caused by trapping of the shelves below the tongue (Fig. 3, second and third rows for all gestational days). By E15.5 the net effect of these perturbations is clefting of the secondary palate with or without associated clefting of the lip/primary palate.

**Figure 4:**
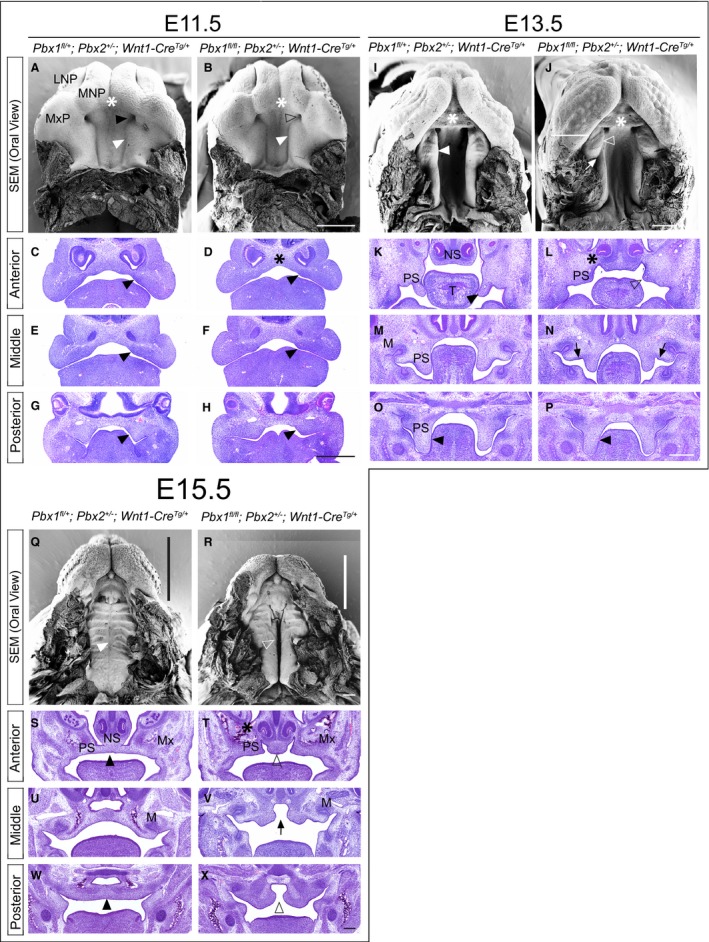
*Pbx* CNCC mutants result in isolated clefting of the secondary palate (cleft palate only; CPO). Scanning electron micrographs of developing palate (SEM; top) and hematoxylin & eosin (H&E) stained coronal sections (bottom) of embryonic palate and oral cavity at E11.5, E13.5 and E15.5. Representative control: *Pbx1* ^*fl/+*^ *;Pbx2* ^*+/−*^ *;Wnt1‐Cre* ^*Tg/+*^ (left) and mutant: *Pbx1* ^*fl/fl*^ *;Pbx2* ^*+/−*^ *;Wnt1‐Cre* ^*Tg/+*^ (right) embryos for each time point. E11.5: SEM shows comparable morphology of nascent primary (white asterisk) and secondary (white arrowhead) palate in control (A) and mutant (B). In mutant, smaller embryonic choanae (empty black arrowhead in B) vs. control (black arrowhead in A) and distance between palatal shelf primordia subtly constricted (B). H&E confirms grossly comparable morphology of nascent palatal shelves in control and mutant (black arrowheads in C–H), with mildly abnormal dorsal aspect of forming nasal septum in mutant (black asterisk in D). E13.5: SEM shows normal formation of primary palate (white asterisk in I,J) in control and mutant with profound dysmorphology of mutant secondary palate, which exhibits aberrant organization of its A–P domains separated by abnormal groove (white arrow in J). Specifically, in mutant, anterior palatal domain bearing rugae (presumptive hard palate) (white empty arrowhead in J) is positioned more laterally and posteriorly than in control (white arrowhead in I). H&E highlights markedly hypoplastic mutant anterior palatal shelves (black empty arrowhead in L) vs. control (black arrowhead in K). Aberrant A–P organization of mutant secondary palate results in abnormal connection between nasal septum and maxilla (black asterisk in L). Sections of middle palate demonstrate presence of epithelial thickenings (black arrows in N) consistent with presence of rugae in more posterior domains of mutant shelves as compared with control (M). Posterior presumptive soft palate comparable in control and mutant (black arrowheads in O, P). E15.5: SEM illustrates reduced outgrowth of midfacial complex in mutant vs. control (compare length of black and white bars along A–P snout in Q and R, respectively). Lack of palatal shelf fusion evident in mutant (white empty arrowhead in R) as compared with control (white arrowhead in Q). H&E demonstrates that in mutant vestigial anterior palatal shelves do not make contact medially (black empty arrowhead in T) vs. control (black arrowhead in S). In addition, in mutant, abnormal tissue connection between nasal septum and maxilla persists (black asterisk in T). Mid‐palatal sections show dysmorphic palatal shelves and clefting at midline in mutant (black arrow in V). Posterior palatal sections demonstrate clefting of soft palate (empty black arrowhead in X) as compared with control (black arrowhead in W). LNP, lateral nasal process; MNP, medial nasal process; M, molar tooth bud; Mx, maxilla; MxP, maxillary process; NS, nasal septum; PS, palatal shelf; T, tongue. Scale bar: 400 μm.

Whereas excision of *Pbx1* from the cephalic epithelium results in lip and palatal clefting phenotypes, excision from premigratory CNCC‐derived mesenchyme, on a *Pbx2*‐deficient mixed genetic background, yields a strikingly different phenotypic outcome (Fig. 4 and Supporting Information Fig. S8). At E11.5, SEM and histological analyses demonstrated that control and mutant embryos were largely comparable as far as their gross morphology. Two subtle morphological perturbations observed at this gestational day include a reduction in size of the embryonic choanae and a narrowing of the inter‐palatal shelf distance (Fig. 4A,B). However, by E12.5, marked morphological abnormalities were obvious in the developing anterior secondary palate (Fig. S8A,B). In controls, outgrowth of the midfacial complex between E11.5 and 12.5 can be visualized by the increasing separation of the primary palate from the secondary palate, via the interposition of an anterior palatal domain that will form the presumptive hard palate adjacent to the nasal choanae. This domain expands uniformly along the medial aspect of the elongating secondary palatal shelf (Figs 4A,B and S8A,B). In wild type embryos, a transient groove, visualized by SEM, normally separates the presumptive hard palate from the presumptive soft palate at E12.5. Anterior to this groove will be the formation of the first palatal ruga (Welsh & O'Brien, 2009). In CNCC mutants, by E13.5, there is a marked exaggeration of this groove, which abnormally persists, and a lateral displacement of the presumptive hard palate, with consequent severe dysmorphology of the secondary palatal shelf (Fig. 4I,J). Of note is the striking resemblance between this phenotype and the reported palatal phenotype in *Satb2*‐null mice (Britanova et al. 2006; Dobreva et al. 2006). As a consequence of the lateral positioning of the presumptive hard palate, the forming mutant soft palate fails to be displaced posteriorly and maintains aberrant proximity to the primary palate. This causes, at least in part, a reduction of the embryonic choanae as compared with controls (Figs 4A,B,I,J and S8A,B,I,J). From E12.5 onwards, the abnormal positioning of the presumptive soft palate in relation to the primary palate and developing sinus cavities results in a bridge of tissue that extends between the lateral aspect of the nasal septum and the ipsilateral maxillary primordium. This aberrant bridge functionally separates the oral and nasal cavities in CNCC mutants, as visualized in H&E‐stained coronal sections (Figs 4K,L,S,T andS8C,D,K,L), similar to the phenotype observed in *Tbx22*‐null mice (Pauws et al. 2009). From E12.5 to E15.5, the mutant anterior palatal shelves beneath this bridge are markedly hypoplastic, whereas in CNCC mutants, clefting of the secondary palate is complete along the A–P axis, by E15.5 the posterior palatal shelves show evidence of medially directed growth (Fig. 4W,X). Interestingly, the CNCC mutant midface also appears overall shorter and wider along the rostro‐caudal axis (Figs 4 andS8).

### *Pbx* CNCC mutants present secondary palate clefting and more severe A–P disorganization of the craniofacial skeleton compared with epithelial mutants

**Figure 5:**
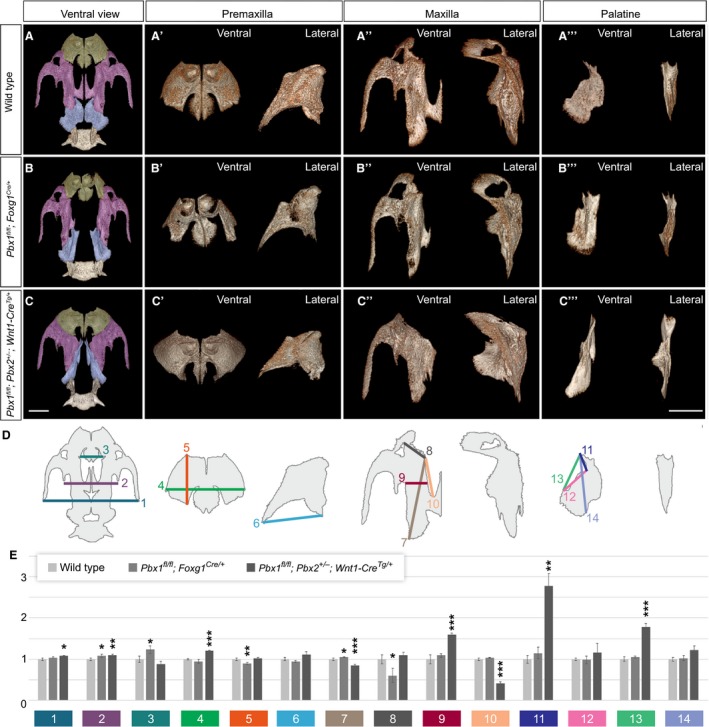
Tissue‐specific deletion of *Pbx* genes results in distinct clefting defects with CNCC‐specific loss concomitantly causing altered positioning and proportions of midfacial skeletal elements. Morphometric analysis of premaxilla, maxilla, and palatine bones in E18.5 wild type and mutant embryos with epithelial‐ and CNCC‐specific loss of *Pbx* genes. (A–C) Ventral μCT reconstructions of palatal regions showing premaxilla (green), maxilla (purple) and palatine bones (pale blue). Ventral and lateral views of individual bones shown in (A′–A′′′) wild type, (B′–B′′′) *Pbx1* ^*fl/fl*^ *;Foxg1* ^*Cre/+*^ and (C′‐C′′′) *Pbx1* ^*fl/fl*^ *;Pbx2* ^*+/−*^ *;Wnt1‐Cre* ^*Tg/+*^ embryos. In lateral views, ventral side oriented towards the right. (D) Diagram of wild type palatal region illustrating location of individual measurements quantified in (E). (E) Morphometric measurements of palatal structures in *Pbx1* ^*fl/fl*^ *;Foxg1* ^*Cre/+*^ (medium gray; *n* = 3) and *Pbx1* ^*fl/fl*^ *;Pbx2* ^*+/−*^ *;Wnt1‐Cre* ^*Tg/+*^ mutants (dark gray; *n* = 3) relative to wild type controls (light gray; *n* = 4). Colors and numbers correspond to those used in diagram shown in (D). Compared withd type and epithelial‐specific loss of *Pbx1*, CNCC mutants exhibit significant shortening and widening of maxilla (C′′; see measurements 7,9,10). In addition, this mutant displays abnormally elongated structure comprising palatal process of maxilla fused to palatine bone (C′′′; palato‐maxillary process, see measurements 11,13). Data are reported as averages ± SEM. Statistical significance: *P ≤ 0.05; **P ≤ 0.01; ***P ≤ 0.001. Scale bars: 1 mm.

We have integrated comprehensive μCT‐based 3D imaging with morphometry (Fig. 5, Supporting Information Fig. S9, Supplementary Table S2, S3, S4) and genetic analyses of E18.5 control and mutant crania, which has proven to be a powerful approach for the quantitative assessment of phenotypes and for highlighting potential regulatory mechanisms underlying craniofacial bone development (Percival et al. 2014; Ho et al. 2015). Excision of *Pbx1* from the cephalic epithelium resulted in minor, albeit statistically significant, differences in measurements of overall cranial structures and palatal elements, including: (i) widening of the distance between both the premaxillae and maxillae (Fig. 5B, measurements 2 and 3; Supporting Information Table S3); (ii) rostro‐caudal shortening of the premaxilla (Fig. 5B′, measurement 5); (iii) diagonal lengthening of the maxilla (Fig. 5B′′, measurement 7); (iv) reduction of the maxillary width at the junction with the premaxilla (Fig. 5B, measurement 8); (v) increase in the distance from the incisor alveolus to the basisphenoid (Table S4). Interestingly, the morphological changes observed in the maxillary bone are associated with a near‐complete loss of the palatine process of the maxilla (Fig. 5A″,B″), a structure that forms the greater part of the hard palate (Richman et al. 2006). The palatine bone itself shows altered morphology when compared with controls (Fig. 5A′′′,B′′′); however, significant size differences were not detected. Of all the above morphological abnormalities, rostro‐caudal shortening of the premaxilla (Fig. 5B′, measurement 5) was the most significant. Overall, our data establish that *Pbx1* deletion from the cephalic epithelium does not disrupt the overall A–P organization of the midfacial complex and results in CL/P with associated skeletal defects limited to alterations in the premaxillary and maxillary bones.

Excision of *Pbx1* from premigratory CNCC‐derived mesenchyme, on a *Pbx2*‐deficient mixed genetic background, yielded a fully penetrant CPO phenotype, rather than CL/P (Fig. 4), together with a severe disruption of A–P craniofacial skeletal organization (Fig. 5C). In contrast to epithelial mutants, μCT‐based morphometry highlighted major differences in measurements of overall cranial structures and palatal elements in CNCC mutants. Statistically significant alterations in these mutants include: (i) increase of the intermaxillary distance (Fig. 5C, measurements 1,2); (ii) increase of premaxillary width (Fig. 5C′, measurement 4); (iii) profound shortening and widening of the maxillae (Fig. 5C′′, measurements 7,9,10); (iv) elongation of the palatine bone, as detailed below; (v) rostro‐caudal shortening of the cranial base, as measured from the basioccipital bone to the incisor alveolus (Table S4, measurement q). The overall effect of these perturbations is a dramatic broadening and shortening of the CNCC mutant midface (Fig. 5C, Table S3, S4). In addition, the maxillary malformations in CNCC mutants include a partial occlusion of the infra‐orbital foramen, where the maxillary branch of the trigeminal nerve egresses the cranium (Fig. 5C′′), as well as markedly abnormal caudal palatine structures (Fig. 5C′′′). Specifically, there is a significant rostro‐caudal elongation of the presumptive palatine bone, which is highly dysmorphic and positioned more anteriorly within the midfacial complex. Rostrally, the palatine bone is ectopically fused with a small, elongated structure that, based on morphology and topology, appears to be the palatine process of the maxilla. This ectopic formation, which also exhibits an additional fusion with the overlying vomer, generates a neomorphic mutant skeletal element that we call ‘palato‐maxillary process’ (Fig. 5C,C′′′, Table S3, Supporting Information Movies S1–S3). Lastly, in CNCC mutants the spatial organization of the basisphenoid, pterygoid and palatine bones is altered. Specifically, the pterygoid bone, which is normally located ventral to the basisphenoid, is shifted rostrally in CNCC mutants (Fig. S10C,C′). This malformation has consequences for the functional architecture of the caudal pharyngeal region and the associated musculature. Consequently, these defects could have a deleterious impact on deglutition and mastication.

In summary, our findings establish that loss of PBX function in either the cephalic epithelium or CNCC‐derived mesenchyme results in the orofacial clefting phenotypes CL/P or CPO, respectively. Importantly, the CNCC‐specific perturbation also produces markedly more severe morphological alterations of individual skeletal elements and overall A–P organization of the midfacial complex as compared with the epithelial‐specific mutation. The presence of ectopic bone formation and aberrant fusions in CNCC mutants strongly suggests that PBX TFs function in the CNCC to control the spatiotemporal dynamics of osteoblast differentiation during ossification of the craniofacial skeleton, a hypothesis supported by the role we previously described for PBX1 in endochondral ossification (Selleri et al. 2001; Gordon et al. 2010, 2011).

### Temporally restricted proliferation defects during development of the secondary palate in CNCC mutants are not accompanied by altered apoptosis

**Figure 6:**
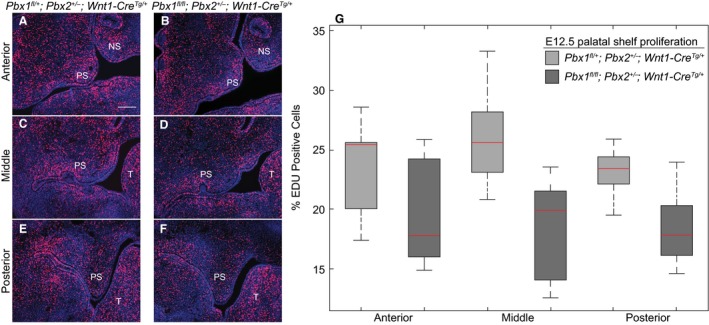
*Pbx* CNCC mutants exhibit proliferation defects along the anterior‐posterior axis of the secondary palate. Proliferation rates assayed by immunofluorescent detection of EdU incorporation in S‐phase cells of developing secondary palate at E12.5. Three pairs of controls: *Pbx1* ^*fl/+*^ *;Pbx2* ^*+/−*^ *;Wnt1‐Cre* ^*Tg/+*^ (left) and mutant: *Pbx1* ^*fl/fl*^ *;Pbx2* ^*+/−*^ *;Wnt1‐Cre* ^*Tg/+*^ (right) littermates processed simultaneously. Sections designated anterior, middle or posterior based on morphological landmarks (left panels, A–F: representative micrographs). Quantification of EdU incorporation presented via box and whisker plots of E12.5 datasets corresponding to anterior, middle or posterior secondary palatal domains (right panel, G). Red bars inside boxes represent median values. At this gestational time‐point, mutant embryos exhibit significant reduction in proliferation rates in all A–P palatal domains. Moderate differences in proliferation between control and mutant at E11.5 (see Fig. S10) presage significantly reduced proliferation rate observed in mutant at E12.5. PS, palatal shelf; NS, nasal septum; T, tongue. Scale bar: 200 μm.

PBX TFs play integral roles in the regulation of mesenchymal progenitor cell proliferation (Brendolan et al. 2005) and subsequent differentiation (Gordon et al. 2011; Hurtado et al. 2015). Based on our observations that in CNCC mutants the anterior palatal shelves appear hypoplastic and dysmorphic starting at E12.5, we examined proliferation rates within the shelves and adjacent maxillae during early‐to‐mid palatogenesis (E11.5–E13.5). Specifically, we carried out detection of EDU‐labeled S‐phase cells (Buck et al. 2008) on serial coronal sections of secondary palates in control and mutant littermates (*n* = 3 per genotype, per time‐point). Shelves were assigned to one of three anatomic levels along the rostro‐caudal axis based on stereotypical morphology. In control palates, we observed a progressive reduction in the mitotic index from E11.5 to E13.5 from approximately 30 to 20% of EdU‐positive cells (Fig. 6A–G, Supporting Information Fig. S11A,B). Whereas at E11.5, proliferation rates in the developing secondary palate of CNCC mutants were only moderately reduced as compared with controls (Fig. S11A), at E12.5 the proliferative defect became pronounced in the mutants, which displayed around 20% of EdU‐positive cells vs. 25% in controls (Fig. 6G). However, by E13.5, differences of proliferation were no longer evident in mutants compared with controls, in both of which approximately 20% of mesenchymal cells were EdU‐positive (Supporting Information Fig. S11B). These results are consistent with a precocious reduction of cell proliferation in CNCC mutant palatal progenitor cells.

PBX loss is not typically associated with increased apoptosis *in vivo* and, indeed, absence of PBX TFs results in localized suppression of apoptosis at the site of facial prominence fusion in the mouse embryo (Ferretti et al. 2011). However, it has been reported that PBX loss from specific cellular populations (Murphy et al. 2010; Grebbin et al. 2016) results in impaired cell survival. For this reason, we examined programmed cell death in the developing secondary palate from early‐ to mid‐palatogenesis (E11.5–E13.5) (Fig. S11C). Immunofluorescent Ab staining for activated Caspase‐3 on serial coronal sections revealed no detectable differences in numbers of Caspase‐3‐positive cells between controls and mutant palates at all time‐points examined, with negligible apoptotic cells in either genotype (Fig. S11C).

### Normal patterning but altered positioning of the presumptive anterior and posterior secondary palatal domains in *Pbx* CNCC mutant embryos visualized by marker gene expression

**Figure 7:**
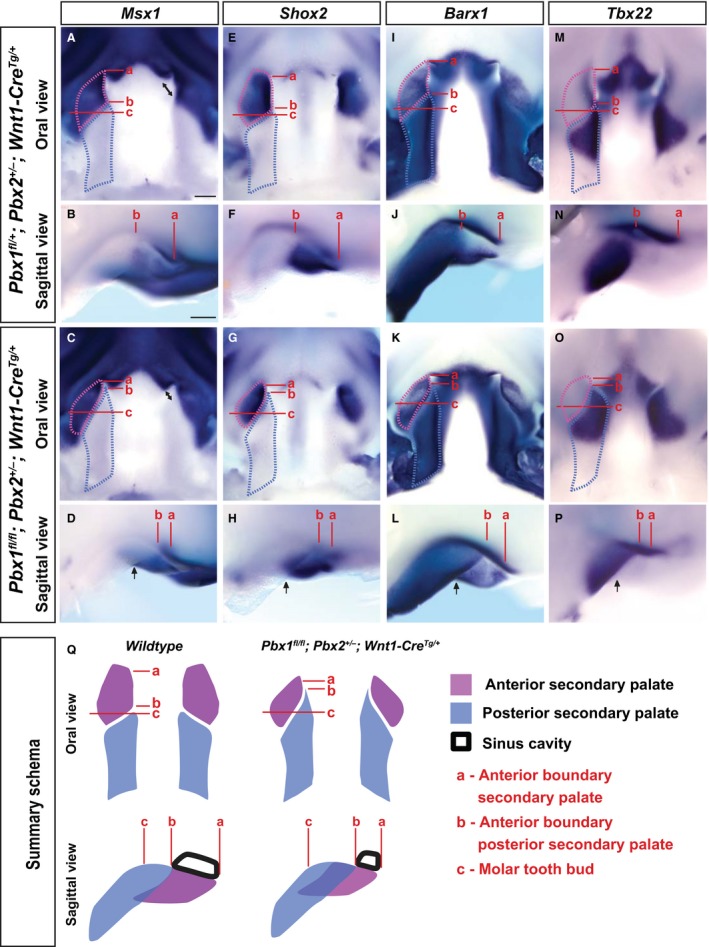
Altered positioning of transcriptional domains along the palatal shelf anterior‐posterior axis in *Pbx* CNCC mutant embryos. WISH reveals that spatial expression of anterior palate markers: *Msx1* (A–D); *Shox2* (E–H); epithelial‐*Barx1* (I–L)*,* as well as posterior palate markers: mesenchymal‐*Barx1* (I–L) and *Tbx22* (M–P), is perturbed in E12.5 CNCC mutants vs. controls (anterior is to the top in oral views and to the right in sagittal views). Anterior boundary of (a) anterior secondary palate, (b) posterior secondary palate and (c) molar tooth bud. All gene transcripts detectable at comparable levels in control (top) and mutant (middle) palates; however, spatial expression domains in anterior vs. posterior secondary palates show altered organization along A–P axis in mutants as compared with controls. Length of posterior secondary palate is similar between controls and mutants (light‐blue dashed outline), whereas the length of mutant anterior secondary palate is slightly reduced (light purple dashed outline; compare distance a–c). Significantly, rather than being positioned rostral to the posterior secondary palate as in controls (distance a–b > b–c), anterior secondary palate in mutants is more caudal (distance a–b < b–c) and lateral (black arrows) to posterior secondary palate. Maxillary dysmorphology accompanied by defective elongation of sinus cavity and nasal septum (black double‐headed arrows in oral view of *Msx1* expression). Morphogenetic and anatomical defects in CNCC mutants summarized schematically (bottom row; Q). Consistent with highly coordinated outgrowth of the midfacial complex, positioning of anterior secondary palate (light purple) rostral to posterior secondary palate (light blue) is perturbed in mutants resulting in cleft palate and consequent reduced expansion of sinus cavity. Scale bar: 200 μm.

CNCC mutants exhibit significantly altered morphology and A–P organization of individual skeletal elements of the upper jaw, which is responsible for the shorter and wider mutant midfacial complex. Many studies have established that distinct regulatory networks are responsible for the development of the anterior and posterior regions of the secondary palate (Hilliard et al. 2005; Okano et al. 2006; Gritli‐Linde, 2007; Kousa & Schutte, 2016), which give rise to its osseous and muscular components, respectively. To determine whether loss of PBX in CNCC results in altered A–P patterning of the secondary palate, we assessed the spatial expression of a number of marker genes that define the presumptive anterior and posterior palatal fields in E12.5 control and CNCC mutant embryos (Fig. 7). *Msx1*, an early marker of anterior secondary palate, is present in the maxillary component of the first branchial arch (BA1) starting at E9.5 and later in the developing lip, primary palate and anterior secondary palate (Hill et al. 1989). Loss of *Msx1* results in CPO in mice (Satokata & Maas, 1994) due to a proliferation defect in the anterior palatal mesenchyme (Zhang et al. 2002). Furthermore, variants in *Msx1* are associated with non‐syndromic forms of CL/P in humans (Yu et al. 2017). At E12.5, we observed comparable levels of *Msx1* expression in the upper lip and primary palate of control and mutant embryos (Fig. 7A–D). However, in the CNCC mutant anterior secondary palate, *Msx1* expression domain was smaller than in controls and did not extend to the midline; rather it was localized laterally to the groove separating the presumptive hard (anterior) from the soft (posterior) secondary palate (Fig. 7A–D). The perturbation of *Msx1* expression patterns in CNCC mutant palates closely mirrors the dysmorphology observed by SEM (see also Fig. S7A,B).

We next assessed the expression pattern of short stature homeobox gene *Shox2* (Blaschke et al. 1998). Loss of *Shox2* from the anterior palate results in proliferation defects confined to the anterior palatal mesenchyme, yielding CPO (Yu et al. 2005). *Shox2* expression initiates at E11.5 only in the mesenchyme of the anterior palate and remains exclusively localized to the presumptive hard palate of mice (Sun et al. 2013), whereas in humans it is detected from Carnegie Stage (CS) 13 onwards (Clement‐Jones et al. 2000). At E12.5, we observed expression of *Shox2* restricted to the anterior domain of the CNCC mutant palate as in controls (Fig. 7E–H). However, recapitulating the perturbed pattern of *Msx1*, in CNCC mutant palates *Shox2* expression domain was laterally constricted and failed to extend medially and anteriorly to the presumptive posterior palatal shelf (Fig. 7E–H).

Because we observed a proliferation defect spanning the entire secondary palate at E12.5, and given the altered anatomic relationship between the anterior and posterior palatal domains in our CNCC mutants, we examined the expression of known markers of posterior palatal fate. BarH‐like homeobox 1 (*Barx1*) is expressed in both the anterior and posterior palatal shelves with restricted epithelial localization only to the anterior domain. Mesenchymal *Barx1* expression in the posterior palate complements the anterior expression patterns of *Msx1* (Welsh & O'Brien, 2009). In CNCC mutants, loss of PBX TFs from the CNCC‐derived mesenchyme did not result in marked alteration of the spatial expression of *Barx1*; however, its anterior boundary remained in closer proximity to the primary palate as compared with controls (Fig. 7I–L). Moreover, in CNCC mutant secondary palates, similar to the perturbed expression of *Msx1* and *Shox2*, the anterior epithelial expression domain of *Barx1* was lateralized vs. controls (Fig. 7I–L). Loss of *Tbx22*, which exhibits restricted expression in the rostral‐half of the presumptive posterior (soft) palate (Fuchs et al. 2010), causes clefting of the posterior soft palate and morphological alterations of the palatine bones (Pauws et al. 2009). In CNCC mutants, *Tbx22* mRNA spatial levels did not appear significantly altered as compared with controls, but the spatial domain and posterior boundary of its expression was modestly altered (Fig. 7M–P). Collectively, our expression analysis establishes that specification of the presumptive anterior and posterior secondary palatal domains is correctly established in *Pbx* CNCC mutant embryos, despite their significantly perturbed and dysmorphic A–P positioning (Fig. 7Q).

**Figure 8:**
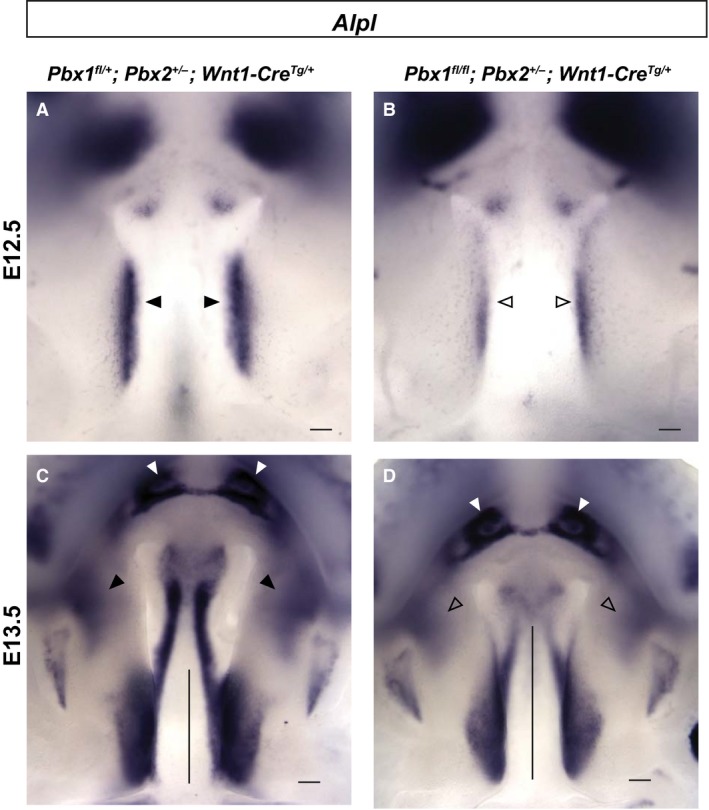
Spatiotemporal dynamics of skeletogenic gene expression is altered in CNCC mutant embryos. Comparison of *Alpl*, a bone alkaline phosphatase, expression in controls and mutants. At E12.5 (A,B) and E13.5 (C,D) *Alpl* expression in posterior palatal shelves is reduced in mutants (open arrowheads) vs. controls (black arrowheads). At E13.5, comparable expression in developing incisors (white arrowheads) of controls and mutants. *Alpl* mRNA levels are reduced overall throughout secondary palate of mutants, in which domains of *Alpl* expression are positioned more laterally in anterior palate (open arrowheads) vs. controls (black arrowheads), and extend rostrally in posterior palate (compare length of vertical black bar in mutant vs. control; Fig. 8C,D). Scale bar: 500 μm.

The findings reported above suggest that loss of PBX in CNCC does not perturb A–P patterning, but rather alters other morphogenetic behaviors (e.g. differentiation, migration, oriented cell division) that drive the formation of the midface. Accordingly, we examined the expression of the *alkaline phosphatase liver/bone/kidney* (*Alpl*) gene, an early marker of skeletal differentiation (Hessle et al. 2002), in control and CNCC mutant embryos. At E12.5, *Alpl* expression, which is restricted to the medial aspect of the posterior palatal shelf, was markedly decreased in mutants vs. controls (Fig. 8A,B). At E13.5, *Alpl* mRNA levels were still overall reduced throughout the secondary palate. However, presaging the skeletal dysmorphology observed at E18.5, in mutants the domains of *Alpl* expression were positioned more laterally in the anterior palate, extended rostrally in the posterior palate, and highlighted shortening and malpositioning of the presumptive vomer (Fig. 8C,D). In summary, our characterization of CNCC mutants shows reduced palate progenitor proliferation; altered A–P positioning of the anterior secondary palate and perturbed skeletal differentiation, demonstrated by marker analyses; and perturbed A–P skeletal patterning including ectopic ossification observed via μCT. All of these findings are consistent with a role for PBX factors in coordinating CNCC‐dependent morphogenesis and skeletal differentiation. Ultimately, the abnormalities caused by CNCC‐specific PBX loss result in reduced elongation and concomitant broadening of the mutant midfacial complex.

## Discussion

In the midface, primary and secondary palate development involves stereotypic morphogenetic processes (outgrowth, proliferation, elevation, fusion; Bush & Jiang, 2012; Lan et al. 2015). The identification of key regulatory factors with restricted spatiotemporal expression that drive reciprocal tissue interactions between cephalic epithelium and CNCC‐derived mesenchyme (Hilliard et al. 2005) is critical to our understanding of how coordinated midfacial development is achieved (Lane & Kaartinen, 2014). Among multiple TFs, our study establishes that PBX homeodomain proteins function in a tissue‐specific manner as well as iteratively to govern the morphogenesis and fusion of the primary and secondary palate.

High levels of PBX1 and PBX2 proteins are present in both cephalic epithelium and CNCC‐derived mesenchyme from early stages of midfacial development, before primary and secondary palatogenesis has occurred and before the facial processes have fully developed, throughout subsequent midfacial morphogenesis. Notably, PBX1 and PBX2 exhibit largely overlapping localization, with PBX2 consistently present at lower levels in primary and secondary palatal domains. This underlies collaborative roles and iterative functions of these two family members in patterning and morphogenesis of the midface, which complements previously reported genetic interactions of *Pbx1* and *Pbx2* in directing the development of multiple organ systems (Capellini et al. 2006, 2008, 2010; Ferretti et al. 2011; Koss et al. 2012;2011 reviewed in Capellini et al. 2011; Golonzhka et al. 2015). Previous studies have shown preeminent roles of PBX factors in the mesenchymal compartments of various organs, including limb (Capellini et al. 2006), axial skeleton (Capellini et al. 2008), spleen (Koss et al. 2012), pancreas (Kim et al. 2002), kidney vascular mural cells and nephrogenic mesenchyme (Hurtado et al. 2015), and lung (Li et al. 2014; McCulley et al. 2018). However, additional evidence also supports important functions for PBX factors in the epithelial compartments of vital organ systems, including the frontonasal processes (Ferretti et al. 2011; Losa et al. 2018) and the pancreas (Kim et al. 2002), as well as ectodermally derived tissues (Golonzhka et al. 2015; Grebbin et al. 2016; Villaescusa et al. 2016).

**Figure 9:**
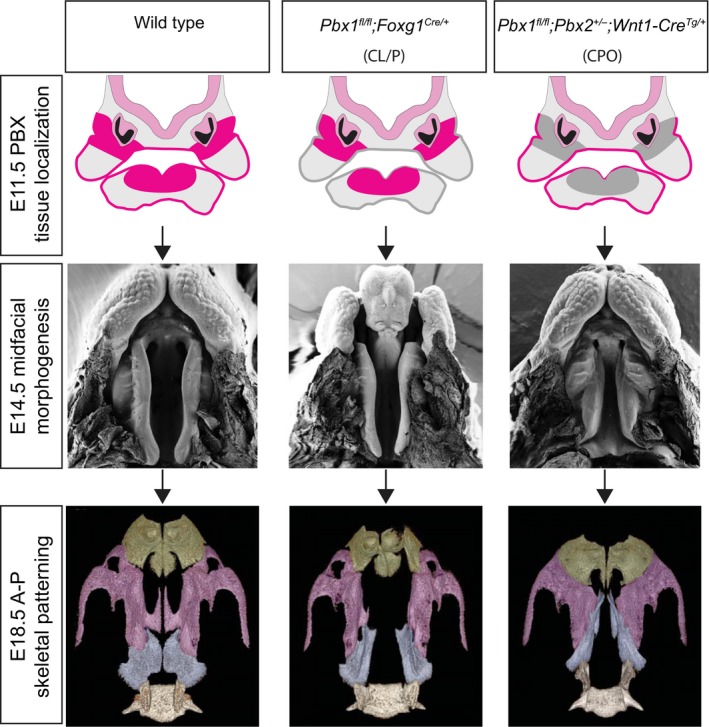
Model for palate morphogenesis in *Pbx* epithelial and CNCC mutant embryos. Top panels: schematics of early expression domains (fuchsia) of *Pbx1* and *Pbx2* in cephalic epithelium and CNCC‐derived mesenchyme of midfacial prominences that will give rise to primary and secondary palate in E11.5 wild type (left), epithelial mutant (middle), and CNCC mutant (right). Domains of tissue‐specific loss of *Pbx* expression highlighted in gray. Middle panels: scanning electron microscopy images of E14.5 wild type (left), epithelial mutant with severe clefting of lip/primary palate and cleft secondary palate (CL/P) (middle), and CNCC mutant with normal lip/primary palate but striking A–P dysmorphology and clefting of secondary palate (CPO; right). Bottom panels: μCT reconstructions of maxillary skeletal element morphology of E18.5 wild type (left), epithelial mutant (middle) and CNCC mutant (right). Premaxilla, maxilla and palatine bones pseudo‐colored in green, purple and pale blue, respectively. Compared with wild type, *Pbx* epithelial‐specific loss results in cleft premaxilla and secondary palate (CL/P) with widening of basisphenoid but unaltered A–P organization of individual skeletal elements. In contrast, *Pbx* CNCC‐specific loss yields clefting of secondary palate only (CPO) accompanied by shortening and widening of maxillary bone and aberrant A–P organization of upper jaw skeletal elements. The altered organization of individual skeletal elements observed at E18.5 is presaged by A–P dysmorphology of secondary palate observed at E14.5 and at earlier developmental stages (see also Figs 4 and Fig. S7).

Consistent with the expression pattern of *Pbx1* and *Pbx2* in the midface, we observed striking but distinct phenotypes in the primary and secondary palate of *Pbx* epithelial and CNCC mutant embryos, respectively (Fig. 9). By studying conditional loss of PBX factors in the cephalic epithelium, here we expanded previous findings on the early roles of these homeodomain proteins in upper lip and primary palate morphogenesis and fusion (Ferretti et al. 2011). Notably, in the present study, only epithelial mutant embryos present CL/P with 67% penetrance. An earlier report in epithelial mutants demonstrated the presence of CL/P with 100% penetrance (Ferretti et al. 2011). However, in that study, embryos were also heterozygous for a constitutive null *Pbx2* allele (*Pbx1* ^fl/fl^;*Pbx2* ^+/−^;*Foxg1* ^Cre/+^), thus sensitizing the genetic background so as to increase the penetrance of this phenotype. In epithelial mutants with unilateral cleft lip, we did observe a direct relationship between clefting of the lip and clefting of the primary palate with only one exception. In contrast, isolated cleft lip is reportedly as high at 42% in some human populations (Elahi et al. 2004), even though it was suggested that individuals diagnosed with CL may have underdiagnosed subclinical palatal defects (Gosain et al. 1999). These findings give weight to the current notion that, whereas in mouse models such variable defects may be interpreted as lack of penetrance, in humans overt clefting phenotypes may be part of a broad morphological spectrum (Marazita, 2012). Here, we also demonstrate a high incidence (95%) of cleft secondary palate in epithelial mutants. However, morphometry shows that these mutants exhibit a broadening of the midface. Given the observed widening of the primary palate at the onset of secondary palate morphogenesis in epithelial mutants, it is not unreasonable to assume that overall disruption of normal midfacial proportions could make clefting of the secondary palate a probable outcome.

Parallel characterization of CNCC mutant embryos, revealed that loss of PBX from CNCC does not result in clefting of the lip or primary palate. Rather, our analysis revealed a later role for PBX factors in morphogenesis of the midfacial complex and secondary palate. Indeed, CNCC mutants exhibit fully penetrant CPO (Fig. 9). CNCC mutants also display strikingly altered A–P positioning of the presumptive anterior and posterior secondary palatal domains and markedly more severe morphological alterations of individual craniofacial skeletal elements than the epithelial mutants do. All of the defects observed in CNCC mutant embryos result in greatly perturbed A–P organization and overall broadening of the midfacial complex. Notably, the presumptive palatine bones are dysmorphic and ectopically fused with the presumptive palatine process of the maxilla and overlying vomer, forming a neomorphic structure, which could result from an earlier perturbation of craniofacial skeletal differentiation. An alternative interpretation of this phenotype is that the dysmorphic palato‐maxillary process represents a rostral duplication of the palatine bone, consistent with potential roles of PBX factors as homeotic proteins. However, the absence of accompanying changes in A–P identity of the domains comprising the presumptive secondary palate, as shown by *in situ* hybridization experiments with select gene markers, argue against this interpretation. Overall, all of the described morphological perturbations result in a striking widening and shortening of the CNCC mutant midface.

Whereas in the upper lip and primary palate, apoptosis in the cephalic epithelium is extensive and is PBX‐dependent, no significant alterations of programmed cell death were identified in *Pbx* CNCC mutants as compared with controls from early to mid‐palatogenesis. In contrast, proliferation rates in the developing secondary palate of CNCC mutants were markedly reduced within a restricted developmental time‐window, which may suggest precocious differentiation of mutant palatal progenitor cells. Accordingly, we observed ectopic bone formation in the lateral maxilla, significant reduction of the infra‐orbital foramen partially obstructed by ectopic bone, and an abnormal palato‐maxillary process, as noted above in *Pbx* CNCC mutants, all consistent with a conserved role for PBX factors in governing the dynamics of progenitor renewal vs. differentiation (Selleri et al. 2001; Ficara et al. 2008, 2013; Gordon et al. 2010, 2011; Hurtado et al. 2015) during midfacial morphogenesis. However, in contrast to loss of PBX in the developing axial skeleton, which results in precocious endochondral ossification (Selleri et al. 2001), *Alpl* expression indicates that intramembranous skeletal differentiation appears to be delayed and not precocious in CNCC mutants as compared with controls. As a result, CNCC‐specific PBX loss perturbs morphogenesis and osteoblast differentiation, disrupting normal rostral extension of the midfacial complex in CNCC mutants.

Related to the phenotypes that we described upon tissue‐specific PBX loss, regulation of the morphogenetic events that link primary and secondary palate formation is still poorly understood. This study establishes that PBX factors play critical and distinct tissue‐specific roles in the sequential formation of the midfacial complex. The presence of PBX TFs in both the epithelium and CNCC mesenchyme suggests that additional tissue‐specific cofactors provide context‐dependent functional output to PBX regulation of target genes in these tissues. We envisage two possible models whereby Pbx1 binding to regulatory elements has either a primary instructive role in target gene transactivation *in vivo* or plays a permissive role as a pioneer factor (Sagerstrom, 2004). The second model is in keeping with reports that have proposed PBX and MEIS as ‘poising’ factors that penetrate chromatin and mark specific genes by forming complexes with tissue‐specific proteins/cofactors that will modify the chromatin environment for activation or repression (Berkes et al. 2004; Sagerstrom, 2004; Choe et al. 2009; Grebbin et al. 2016). We therefore propose the latter scenario as a possible mechanism by which PBX factors coordinate the sequential morphogenesis of the primary and secondary palate.

It is interesting to speculate how primary palate morphogenesis may influence secondary palate development as a requirement for the coordination of midfacial growth. In evolutionary contexts, it was reported that variation in the outgrowth of the midfacial complex (comprising premaxilla, maxilla and palatine bones) is driven by species‐specific mechanisms that act following primary palate formation (Young et al. 2014). For example, in avians, midfacial length is determined primarily by growth of the premaxilla and palatine bones, whereas the intervening maxilla is rudimentary and is associated with obligate clefting of the secondary palate. In contrast, in non‐avians, variation in midfacial outgrowth is determined primarily by differential growth of the maxilla (Young et al. 2014). With direct relevance to human disease, it is notable that studies in human populations have shown the presence of broadening of the faces in patients affected by CL/P (Manyama et al. 2014). In this study, we have characterized two mouse models of orofacial clefting that result from PBX loss‐of‐function in cephalic epithelium or CNCC‐derived mesenchyme, both of which yield CL/P or CPO together with significant widening of the midfacial complex, establishing these mouse strains as unique models to dissect the complexities of orofacial clefting further. Notably, midfacial broadening is more striking in *Pbx* CNCC mutants, which is underpinned by early perturbations in the positioning of A–P secondary palatal domains and resulting marked widening of the maxilla. These findings closely link PBX homeodomain proteins to the variation in maxillary shape and size that occurs in pathological settings, and further suggest possible involvement of these transcription factors in an evolutionary context of midfacial morphological diversity.

## Author contributions

I.W., J.H., J.B., M.G.G. and L.S. contributed to designing, writing and approving the final version of the manuscript. E. F. initiated the project and contributed preliminary data. R.H. and D.H. provided supervision and technical advice to J.H. for the completion of the project after the relocation of L.S. from Weill Cornell to UCSF. I.W., J.H., J.B., K.H., M.R.M., I.G. and R.A. contributed to the acquisition and analysis of the data. All of the authors contributed to the interpretation of the data.

## Supporting information


**Movies S1–S3.** 3D rotational movies rendered using osirix software of palatal region and individual bones isolated with automated osirix 3D Segmentation tool of wild type (Movie S1), epithelial (Movie S2) and CNCC (Movie S3) mutant embryos at E18.5.

**Fig. S1.** Localization of PBX1 in the embryonic head at E10.5.

**Fig. S2.** Localization of PBX2 in the embryonic head at E10.5.

**Fig. S3.** Localization of PBX1 in the developing palate at E13.5.

**Fig. S4.** Unperturbed expression of *Msx1*, a marker of CNCC, in embryos with constitutive loss of *Pbx1/Pbx2*.

**Fig. S5.** Epithelial‐specific loss of *Pbx1* in the developing palate at E13.5.

**Fig. S6.** CNCC‐specific loss of *Pbx1* on a *Pbx2*‐deficient background in the developing palate at E11.5.

**Fig. S7.** Variable penetrance and expressivity of orofacial clefting defects associated with epithelial‐specific loss of *Pbx1*.

**Fig. S8.** CNCC mutants exhibit isolated clefting of secondary palate (cleft palate only; CPO).

**Fig. S9.** Morphometric analysis via landmark positioning highlights more severe craniofacial defects in Pbx CNCC mutants.

**Fig. S10.** Epithelial and CNCC‐specific loss of *Pbx* genes results in distinct abnormal morphologies of the basisphenoid with CNCC mutants presenting altered anterior‐posterior positioning of midfacial skeletal elements

**Fig. S11.** Onset of proliferation defects at E11.5 in CNCC mutants is not accompanied by alteration of apoptosis in the secondary palate.

**Table S1.** Summary of penetrance and expressivity of orofacial clefting defects epithelial‐specific Pbx1 mutants.

**Table S2.** Description of Landmarks and Locations used for morphometric analysis.

**Table S3.** Normalized Palate Landmark Measurement Data for epithelial‐ and CNCC‐specific Pbx1 mutants.

**Table S4.** Normalized Skull Landmark Measurement Data for epithelial‐ and CNCC‐specific Pbx1 mutants..
