# Supplementary figures and images for: Pbx loss in cranial neural crest, unlike in epithelium, results in cleft palate only and a broader midface
Source: J Anat. 2018 May 23;233(2):222–42. doi: 10.1111/joa.12821 (PMC6036936; doi:10.1111/joa.12821)

Supplementary Figure 1.

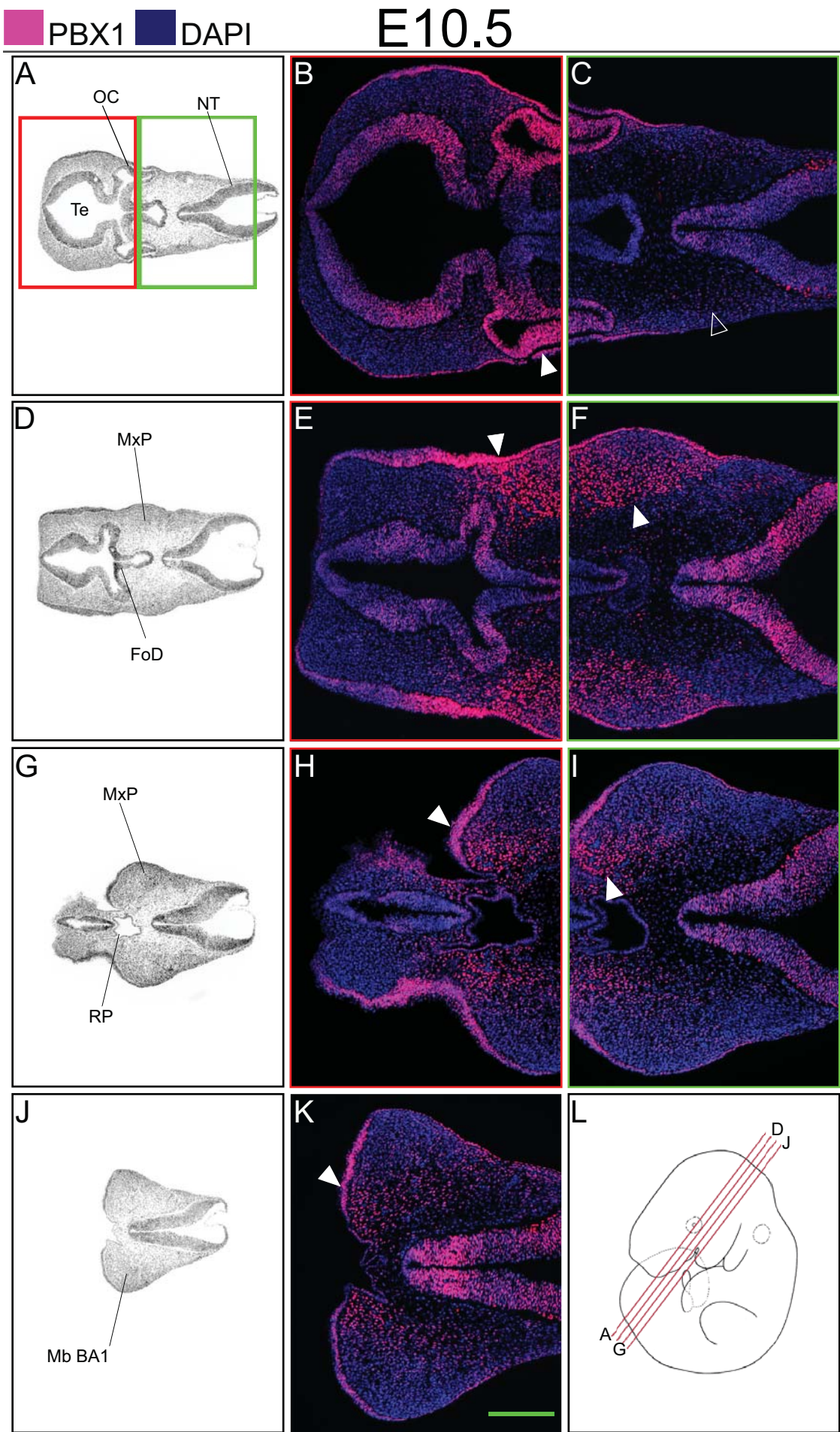

Supplement: Supplementary file 4 — Fig. S1. Localization of PBX1 in the embryonic head at E10.5. [file JOA-233-222-s004.pdf]

Supplementary Figure 2.

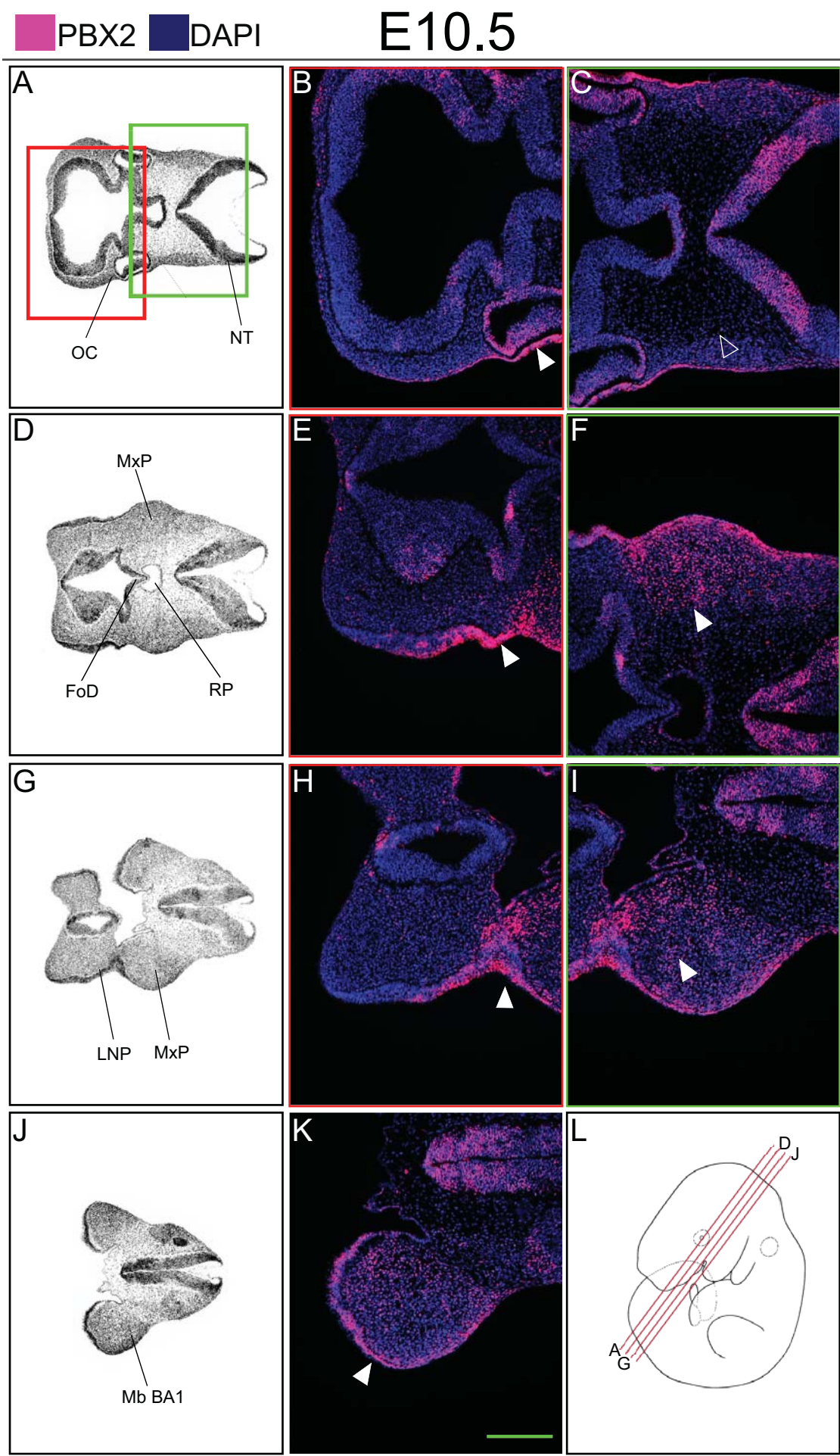

Supplement: Supplementary file 5 — Fig. S2. Localization of PBX2 in the embryonic head at E10.5. [file JOA-233-222-s005.pdf]

Supplementary Figure 3.

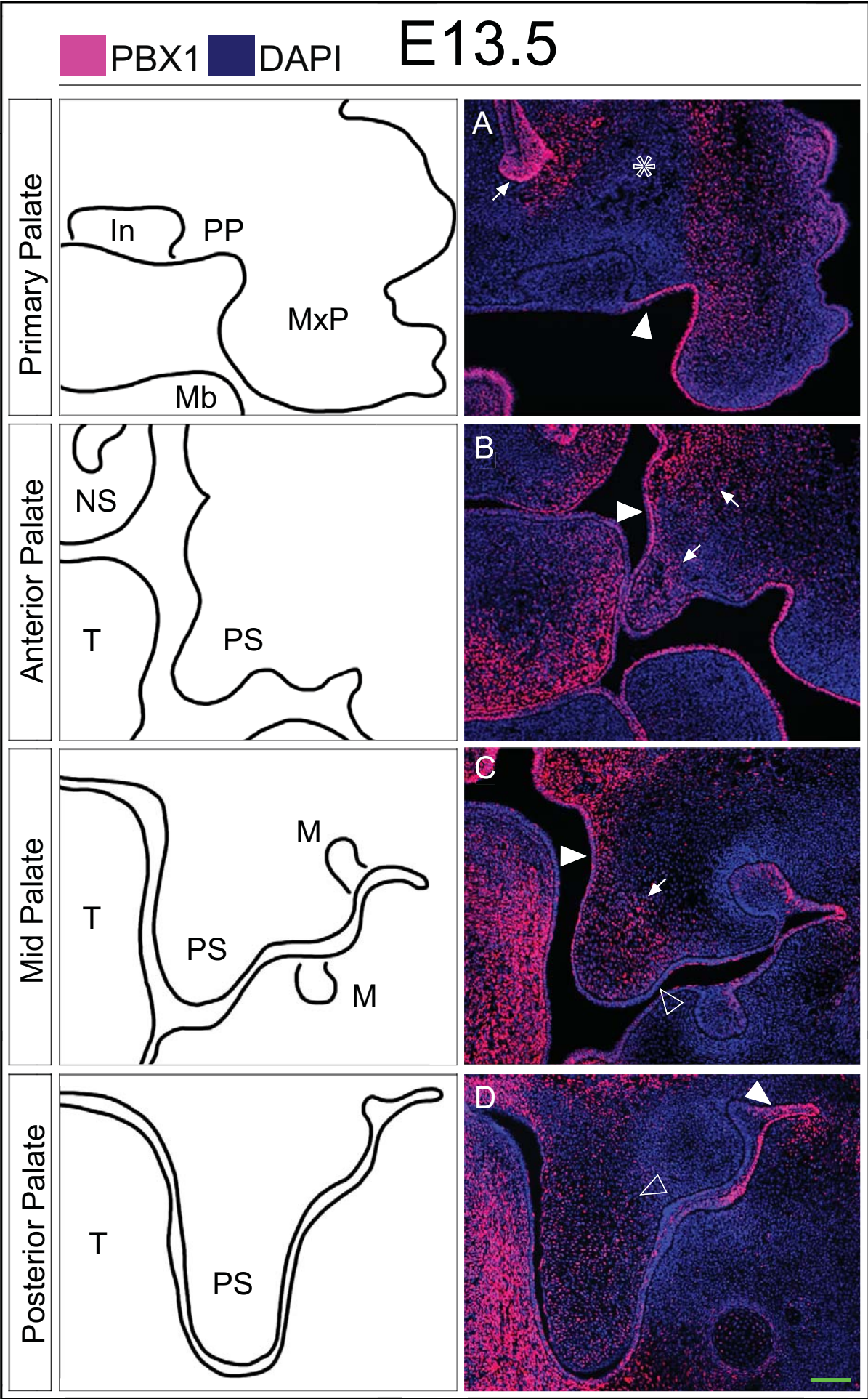

Supplement: Supplementary file 6 — Fig. S3. Localization of PBX1 in the developing palate at E13.5. [file JOA-233-222-s006.pdf]

# Supplementary Figure 4

***Msx1***

***Pbx1*<sup>+/-</sup>;*Pbx2*<sup>+/-</sup>**

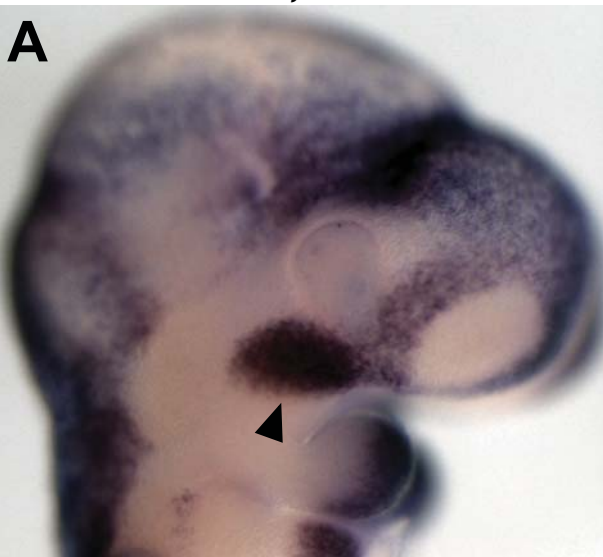

***Pbx1*<sup>-/-</sup>;*Pbx2*<sup>+/-</sup>**

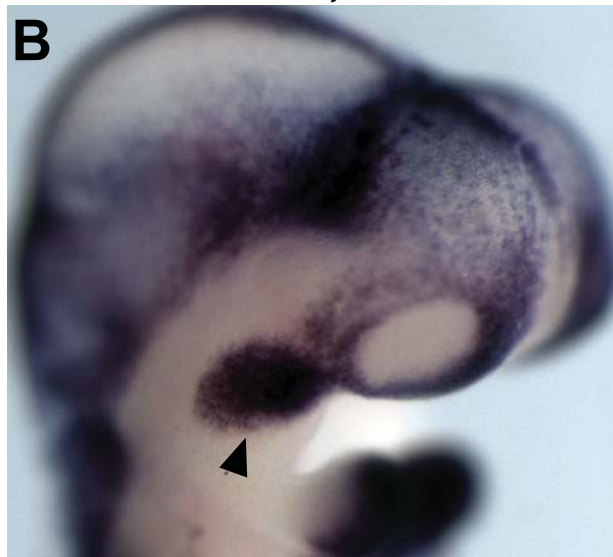

Supplement: Supplementary file 7 — Fig. S4. Unperturbed expression of Msx1, a marker of CNCC, in embryos with constitutive loss of Pbx1/Pbx2. [file JOA-233-222-s007.pdf]

Supplementary Figure 5.

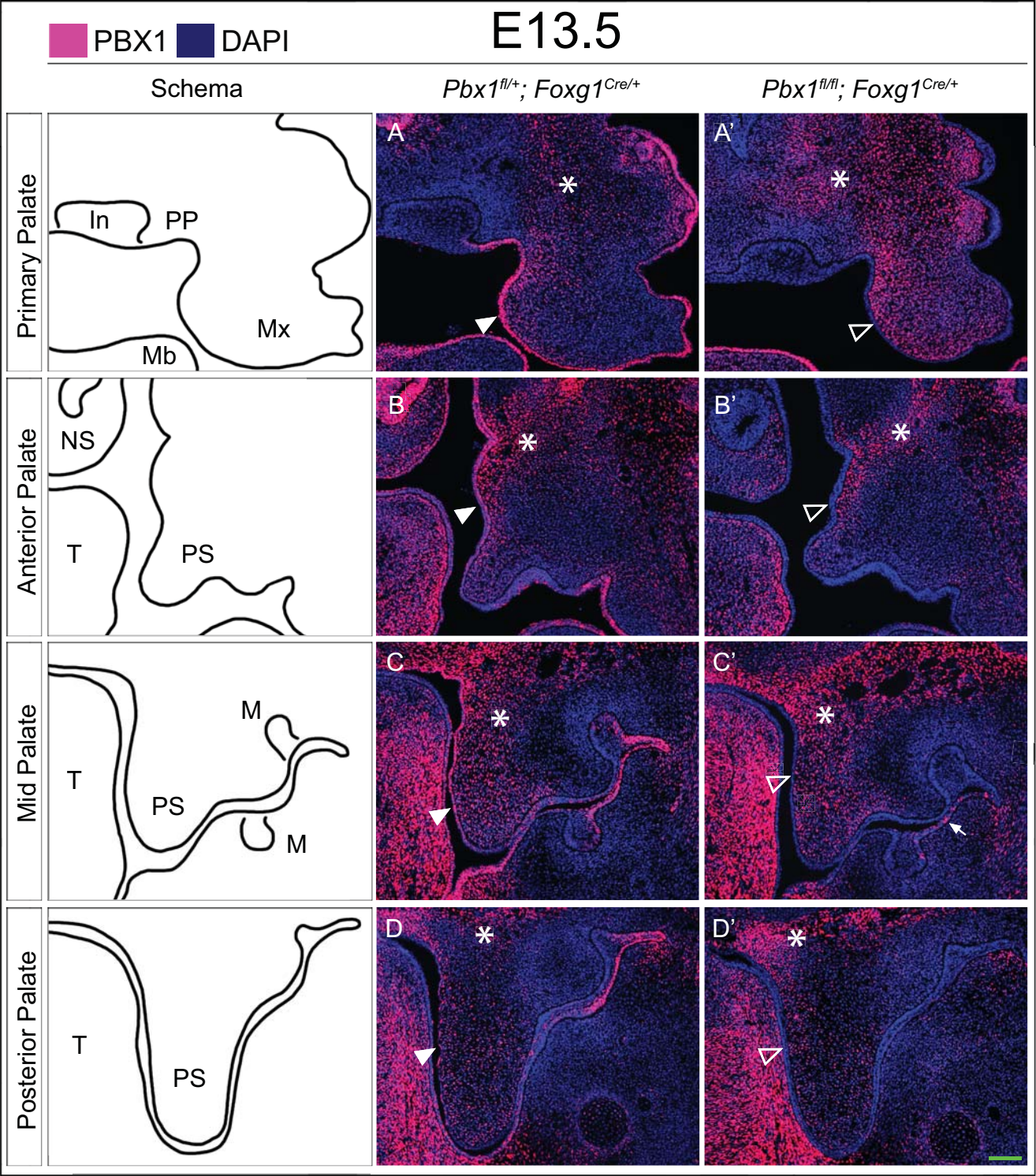

Supplement: Supplementary file 8 — Fig. S5. Epithelial‐specific loss of Pbx1 in the developing palate at E13.5. [file JOA-233-222-s008.pdf]

Supplementary Figure 6.

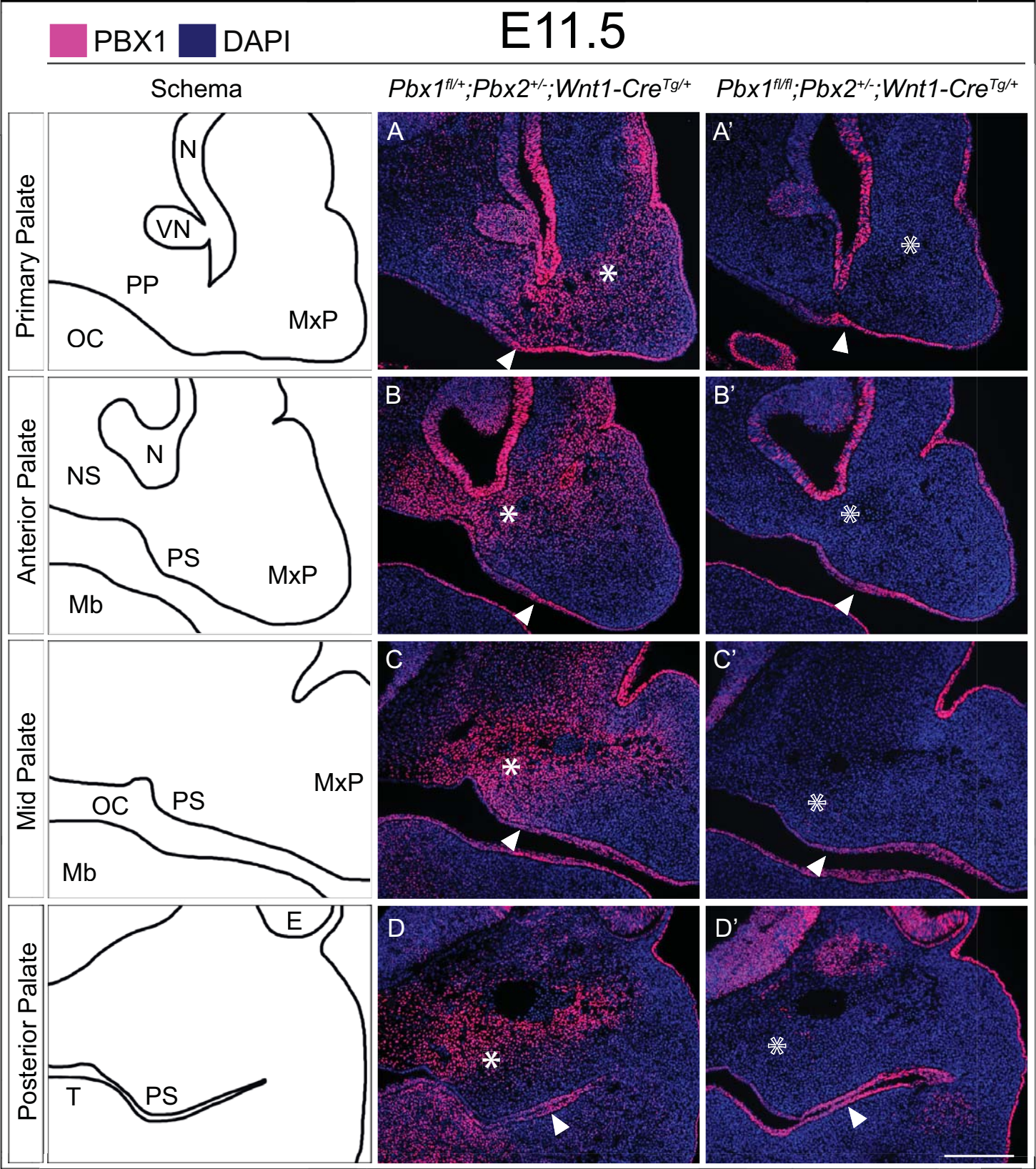

Supplement: Supplementary file 9 — Fig. S6. CNCC‐specific loss of Pbx1 on a Pbx2‐deficient background in the developing palate at E11.5. [file JOA-233-222-s009.pdf]

Supplementary Figure 8.

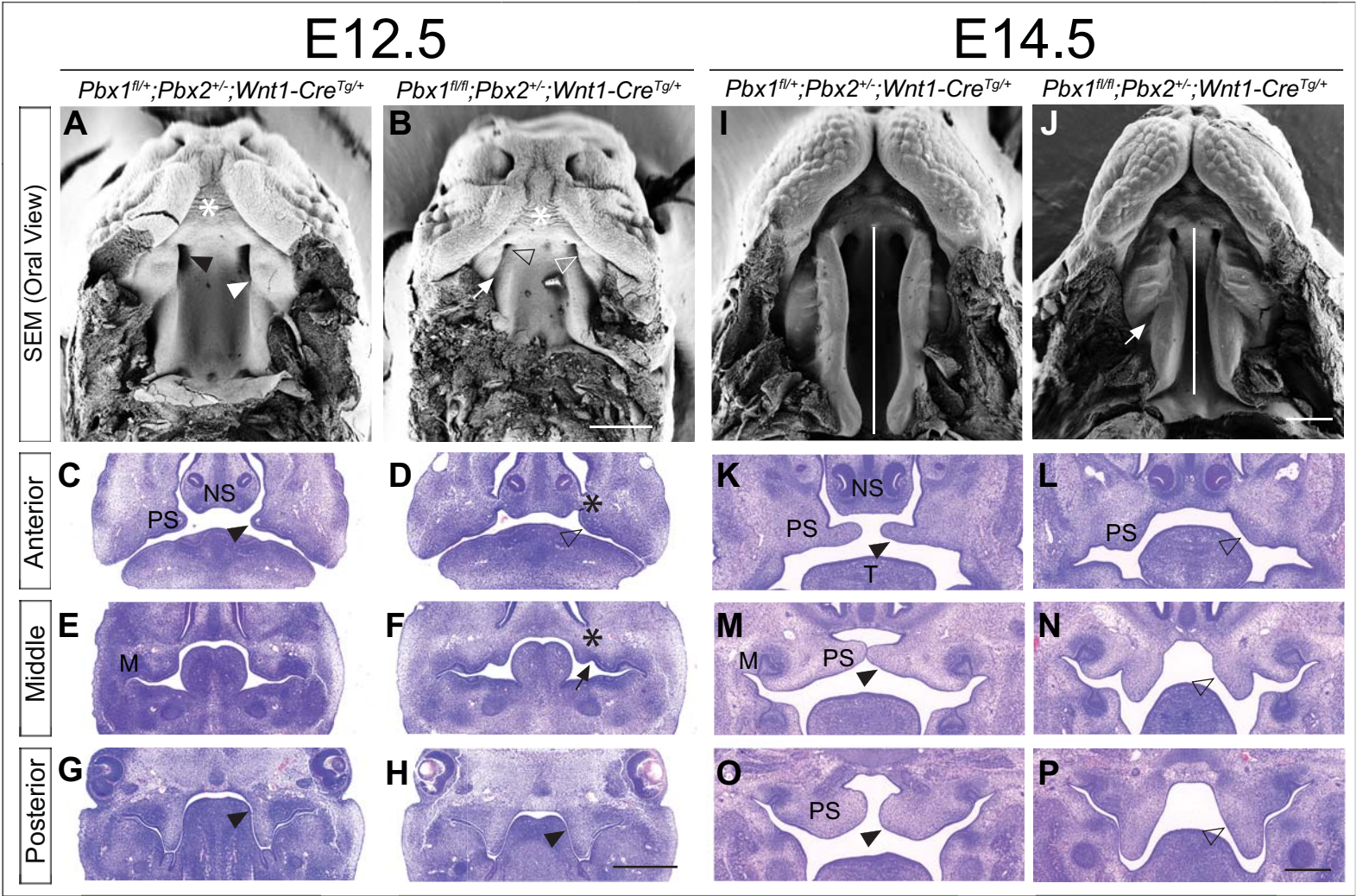

Supplement: Supplementary file 11 — Fig. S8. CNCC mutants exhibit isolated clefting of secondary palate (cleft palate only; CPO). [file JOA-233-222-s011.pdf]

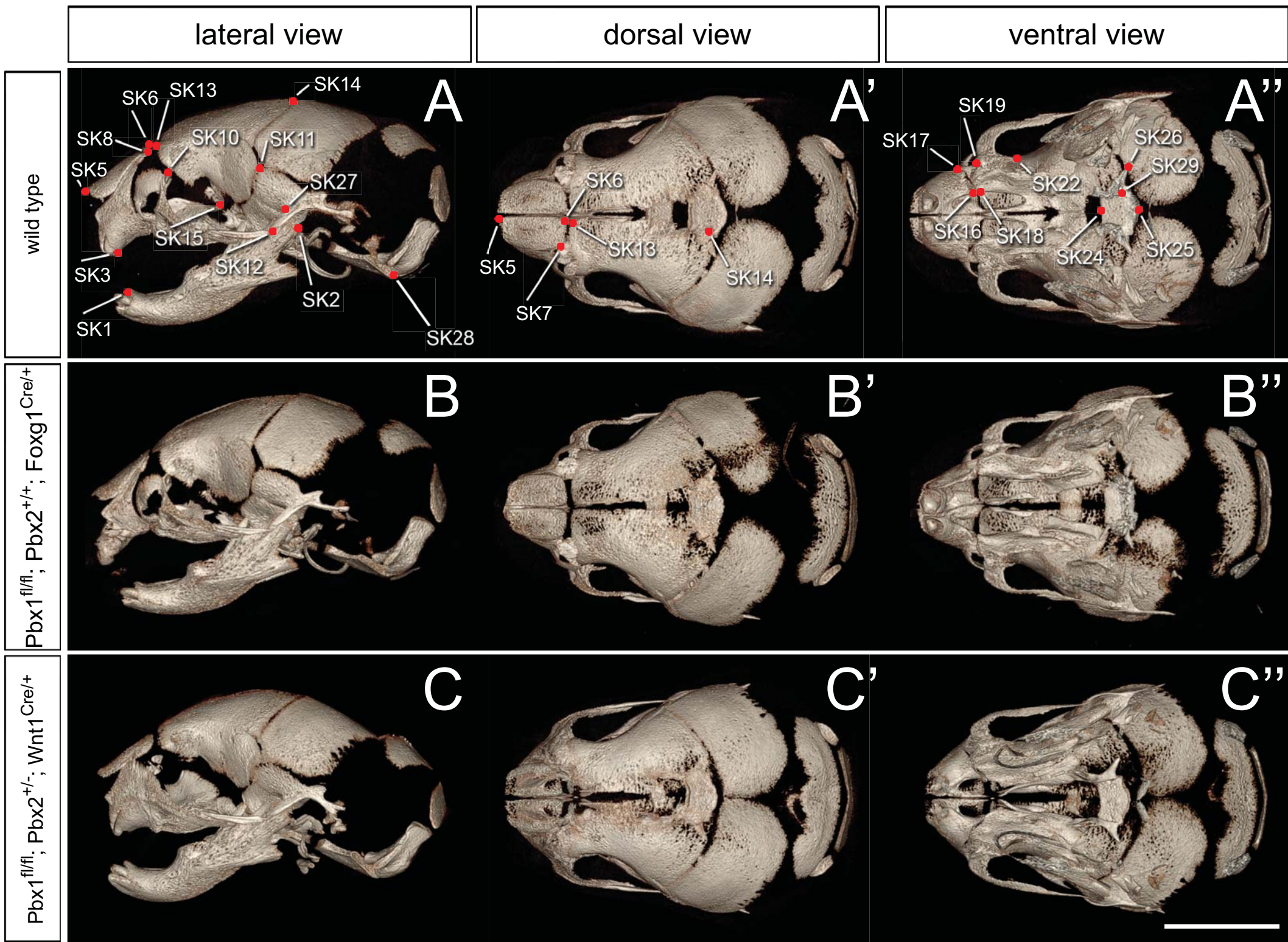

Supplement: Supplementary file 12 — Fig. S9. Morphometric analysis via landmark positioning highlights more severe craniofacial defects in Pbx CNCC mutants. [file JOA-233-222-s012.pdf]

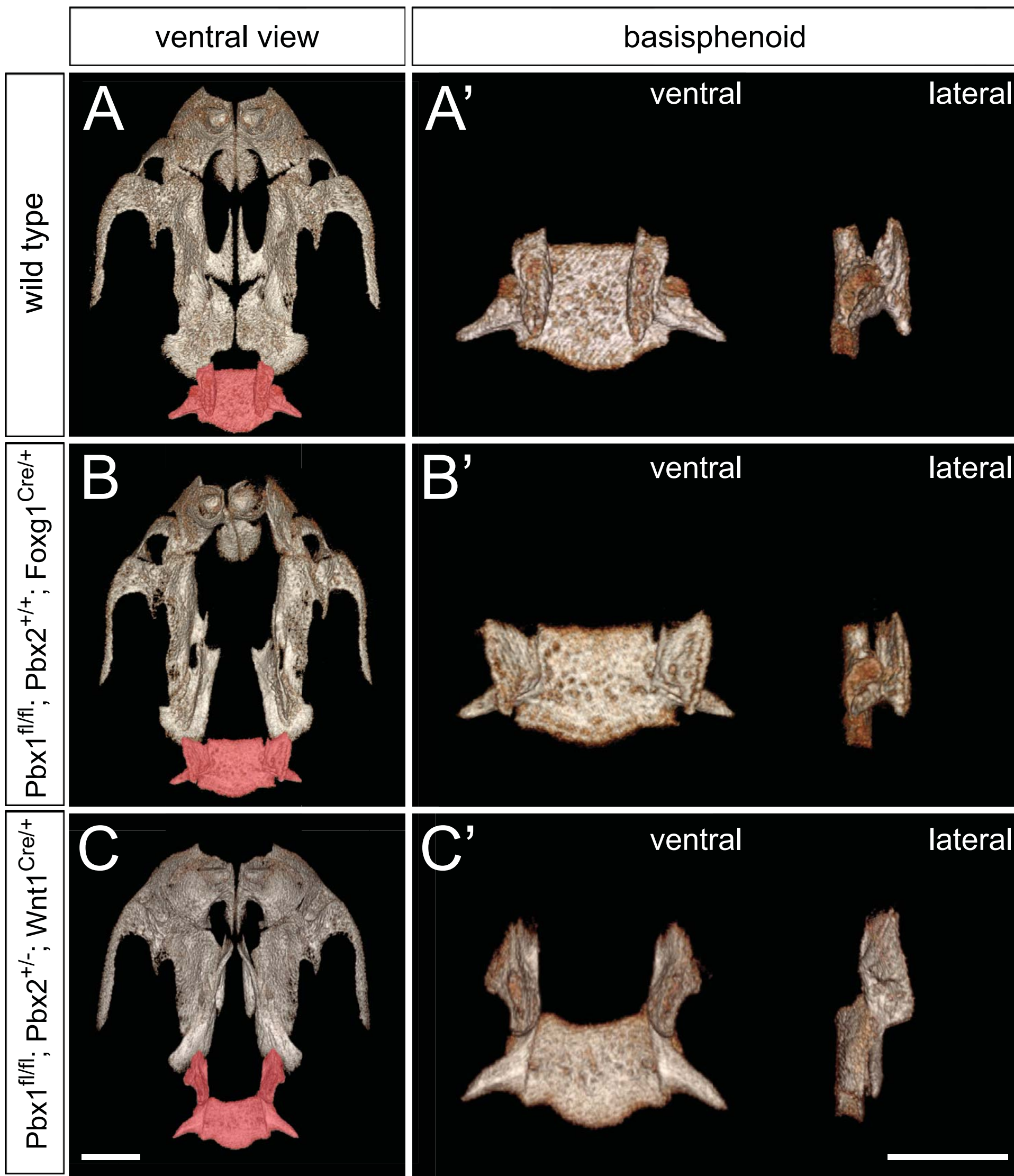

Supplement: Supplementary file 13 — Fig. S10. Epithelial and CNCC‐specific loss of Pbx genes results in distinct abnormal morphologies of the basisphenoid with CNCC mutants presenting altered anterior‐posterior positioning of midfacial skeletal elements [file JOA-233-222-s013.pdf]

Supplementary Figure 11 .

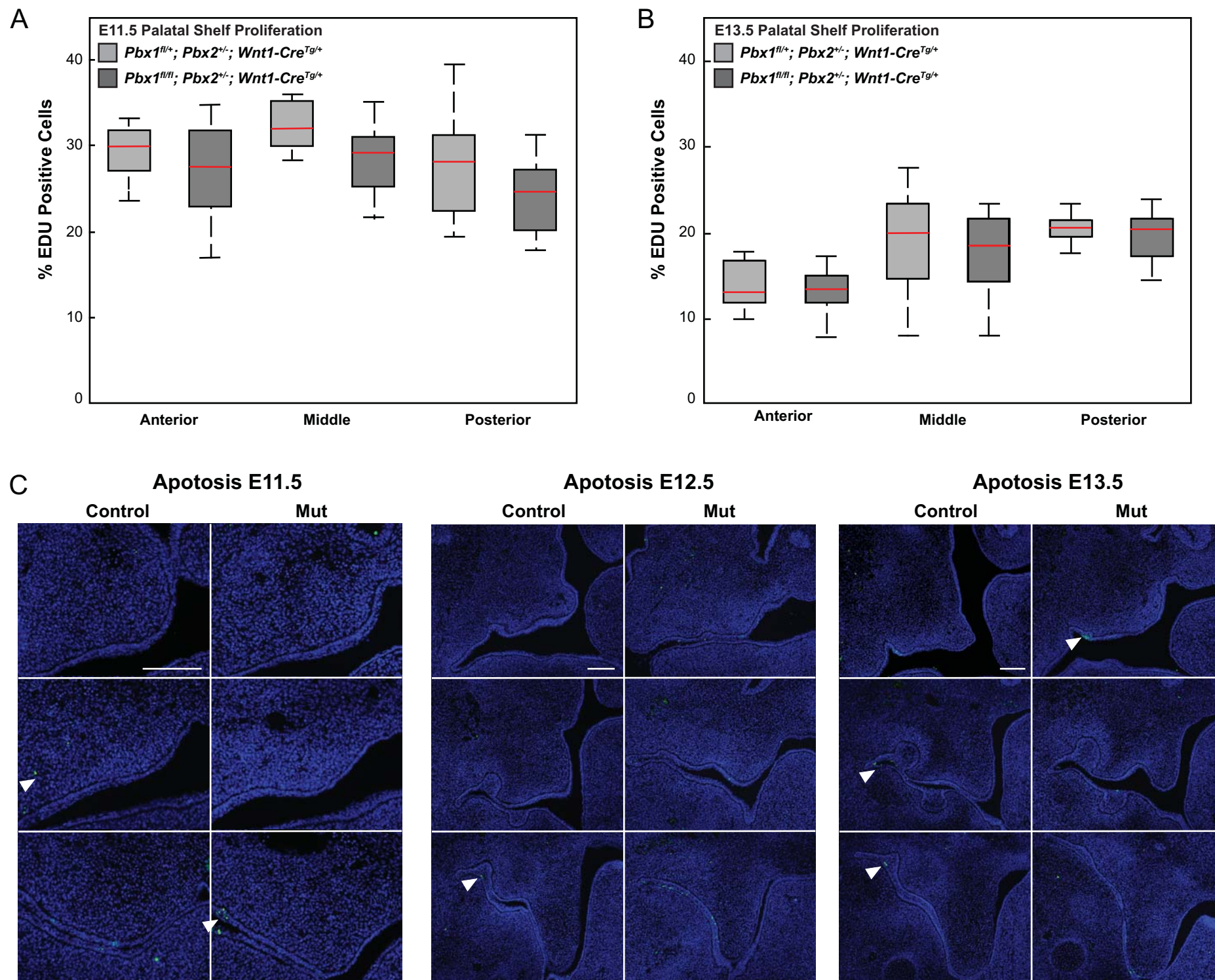

Supplement: Supplementary file 14 — Fig. S11. Onset of proliferation defects at E11.5 in CNCC mutants is not accompanied by alteration of apoptosis in the secondary palate. [file JOA-233-222-s014.pdf]
