# Supplementary material for: Pbx loss in cranial neural crest, unlike in epithelium, results in cleft palate only and a broader midface
Source: J Anat. 2018 May 23;233(2):222–42. doi: 10.1111/joa.12821 (PMC6036936; doi:10.1111/joa.12821)
Supplement: Supplementary file 10 — Fig. S7. Variable penetrance and expressivity of orofacial clefting defects associated with epithelial‐specific loss of Pbx1. [file JOA-233-222-s010.pdf]

Supplementary Figure 7.

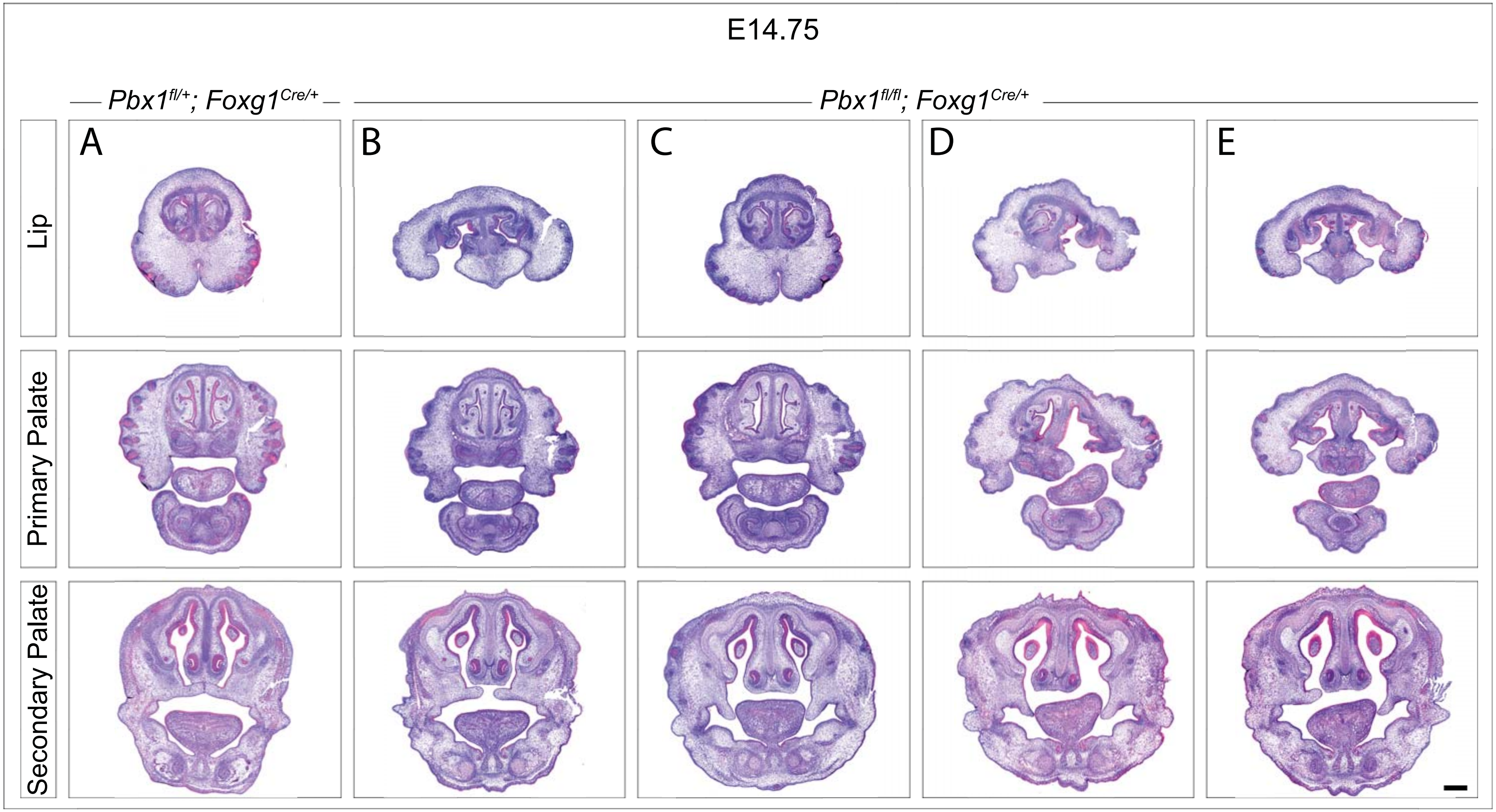

Supplementary Table 1:

	Unitlateral Cleft Lip<br>Normal Primary Palate<br>Normal 2° Palate	Normal Lip<br>Normal Primary Palate<br>Cleft 2° Palate	Unitlateral Cleft Lip<br>Unitlateral CleftPrimary Palate<br>Cleft 2° Palate	Bitlateral Cleft Lip<br>BitlateralPrimary Palate<br>Cleft 2° Palate
N:	1	7	8	5
%:	5%	33%	38%	24%

Total number of $Pbx1^{fl/fl}$; $Foxg1^{Cre/+}$ mutant embryos examined: 21
Total number of litters/embryos* harvested: 11/102
* no craniofacial defects were observed in littermate controls
